# Chemical constituents of *Chaenomeles sinensis* twigs and their biological activity

**DOI:** 10.3762/bjoc.16.257

**Published:** 2020-12-17

**Authors:** Joon Min Cha, Dong Hyun Kim, Lalita Subedi, Zahra Khan, Sang Un Choi, Sun Yeou Kim, Chung Sub Kim

**Affiliations:** 1 School of Pharmacy, Sungkyunkwan University, Suwon 16419, Republic of Korea; 2 College of Pharmacy, Gachon University, #191, Hambakmoero, Yeonsu-gu, Incheon 21936, Republic of Korea; 3 Korea Research Institute of Chemical Technology, Daejeon 34114, Republic of Korea; 4 Gachon Institute of Pharmaceutical Science, Gachon University, Incheon 21936, Republic of Korea

**Keywords:** antineuroinflammation, *Chaenomeles sinensis*, cytotoxicity, megastigmane, neurotrophic effect

## Abstract

A new megastigmane-type norsesquiterpenoid glycoside, chaemeloside (**1**), was isolated from the twigs of *Chaenomeles sinensis* together with 11 known phytochemicals through chromatographic methods. The chemical structure of the new isolate **1** was determined by conventional 1D and 2D NMR data analysis, ECD experiment, hydrolysis followed by a modified Mosher’s method, and LC–MS analysis. The characterized compounds’ biological effects including cytotoxicity against cancer cell lines, antineuroinflammatory activity, and potential neurotrophic effect were evaluated.

## Introduction

*Chaenomeles sinensis* (Thouin) Koehne (Rosaceae) is a deciduous or semi-evergreen tree widely distributed in East Asia including Korea, Japan, and mainland China. The fruits of this medicinal plant have been used on its own or in combination with other medicinal herbs to treat diarrhea, vomiting, myalgia, and common cold [1]. Previous phytochemical studies on this plant have reported triterpenoids [2–8] and phenolic compounds [2,9–12], and some of them showed anti-inflammatory and neuroprotective [13–15], antitumor [2], tissue factor inhibitory [5,9], antibacterial [8], antihemolytic [8], or antipruritic activities [11].

**Figure 1:**
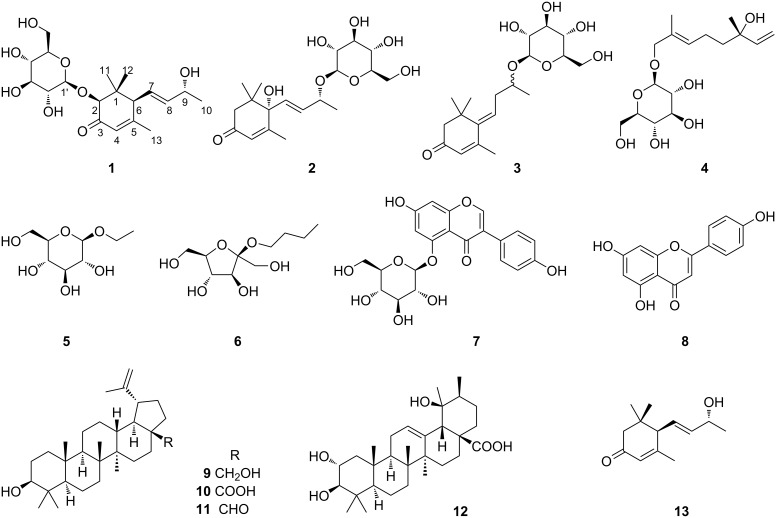
Chemical structures of compounds **1**–**13**.

As a part of the continuing studies to identify bioactive constituents from the Korean medicinal plants [13,16–20], previous phytochemical investigations on the MeOH extract of the twigs of *C. sinensis* have led to the isolation and characterization of triterpenoids [13], biphenyls [14], lignans [15], and oxylipins [21] with cytotoxic, anti-inflammatory, or potential neuroprotective activities. In order to search for minor constituents of other structure classes in *C. sinensis* twigs, the MeOH extract and the four solvent-partitioned fractions were further investigated to afford a new megastigmane-type norsesquiterpenoid glycoside **1** along with 11 known compounds (**2–12**, Figure 1). The structure of the new compound **1** was established on the basis of spectroscopic and spectrometric data analysis, and chiral derivatization coupled with NMR or LC–MS experiments. All isolated compounds **1–12** were evaluated for their cytotoxicity against four human tumor cell lines, antineuroinflammatory activity using lipopolysaccharide (LPS)-stimulated murine microglia BV-2 cell lines, and potential neurotrophic effects in C6 cells.

## Results and Discussion

**Figure 2:**
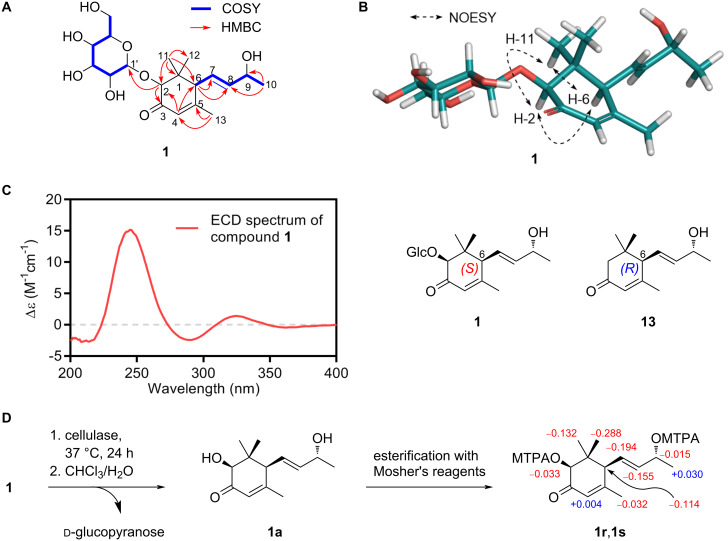
Structure elucidation of compound **1**. (A) Key COSY (blue bold) and HMBC (red arrows) correlations of **1**. (B) Key NOESY (black dashed) correlations of **1**. The 3D structure of **1** was obtained by geometry optimization at the MMFF force field. (C) ECD spectrum of **1** and structures of **1** (6*S*) and **13** (6*R*). (D) Enzymatic hydrolysis of **1** followed by modified Mosher’s esterification of the aglycone **1a**.

From the *n*-BuOH-soluble fraction of the MeOH extract of *C. sinensis*, the new compound **1** was isolated guided by the characteristic TLC spot detected under UV light or by heating after spraying anisaldehyde–sulfuric acid. Chaemeloside (**1**) was obtained as a colorless gum and its molecular formula was determined as C_19_H_30_O_8_ on the basis of the sodiated molecular ion peak at *m*/*z* 409.1830 (calcd for C_19_H_30_O_8_Na^+^, 409.1833, error = 0.7 ppm). The ^1^H and HSQC NMR data of compound **1** suggested the presence of three olefinic protons [δ_H_ 5.96 (dt, *J* = 2.8, 1.5 Hz, 1H), 5.74 (dd, *J* = 15.3, 5.8 Hz, 1H), and 5.58 (ddd, *J* = 15.3, 10.1, 1.1 Hz, 1H)], seven oxygenated methines [δ_H_ 4.41 (d, *J* = 7.8 Hz, 1H), 4.31 (m, 1H), 4.24 (s, 1H), 3.39 (t, *J* = 9.0 Hz, 1H), 3.30 (1H, overlap), 3.29 (1H, overlap), and 3.24 (1H, overlap)], an oxygenated methylene [δ_H_ 3.86 (dd, *J* = 11.8, 2.2 Hz, 1H) and 3.66 (dd, *J* = 11.8, 5.9, 1H)], a methine [δ_H_ 3.04 (d, *J* = 10.0 Hz, 1H)], and four methyl groups [δ_H_ 1.88 (brs, 3H), 1.27 (d, *J* = 6.4 Hz, 3H), 1.15 (s, 3H), and 0.84 (s, 3H)]. The ^13^C NMR of compound **1** showed total 18 resonances, a carbonyl (δ_C_ 200.6), four olefinic (δ_C_ 165.3, 141.9, 126.6, and 125.6), a dioxygenated (δ_C_ 104.9), seven monooxygenated (δ_C_ 87.8, 78.3, 78.2, 75.7, 71.5, 68.7, and 62.9), and six methyl/methylene/methine [δ_C_ 57.2, 43.4, 26.0, 23.8 (×2), and 16.5] carbons. Further investigation of the NMR data (Table 1) suggested the presence of a glucopyranosyl unit in compound **1** with the characteristic ^13^C NMR signals (δ_C_ 104.9, 78.3, 78.2, 75.7, 71.5, and 62.9) and the anomeric proton signal in the ^1^H NMR spectrum [δ_H_ 4.41 (d, *J* = 7.8 Hz, 1H)]. The remaining NMR data of the aglycone were similar to those of a megastigmane-type norsesquiterpenoid, (6*R*,7*E*,9*R*)-9-hydroxy-4,7-megastigmadien-3-one (**13**, Figure 1) [22], with the significant difference of the downfield-shifted NMR resonances at H-2 [δ_C_ 4.24 for **1**; δ_C_ 2.30 and 2.09 for **13**] and C-2 [δ_C_ 87.8 for **1**; δ_C_ 48.4 for **13**] suggesting the presence of a hydroxy group at C-2 in compound **1**. This initial proposal was supported by the HMBC correlations of H-4 and H-11 with C-2 and H-2 with C-3 (Figure 2A). The glucopyranosyl unit was confirmed to be located at C-2 through a glycosidic bond by the HMBC cross-peak between H-2 and C-1′ (Figure 2A). The intensive analysis of the 1D and 2D NMR data of compound **1** including COSY, HSQC, and HMBC analyses led to the elucidation of the planar structure of **1** (Figure 2A and Supporting Information File 1).

**Table 1:** ^1^H [ppm, mult., (*J* in Hz)] and ^13^C NMR data of compounds **1** and **1a** in methanol-*d*_4_.

Position	**1**		**1a**	

	δ_C_	δ_H_	δ_C_	δ_H_

1	43.4		44.4	
2	87.8	4.24, s	81.6	3.96, s
3	200.6		200.9	
4	125.6	5.96, dq (2.8, 1.5)	125.0	5.98, dq (2.7, 1.3)
5	165.3		164.6	
6	57.2	3.04, d (10.0)	56.8	3.02, dq (10.1, 1.7)
7	126.6	5.58, ddd (15.3, 10.1, 1.1)	126.2	5.55, ddd (15.3, 10.2, 1.3)
8	141.9	5.74, dd (15.3, 5.8)	142.0	5.75, dd (15.3, 5.9)
9	68.7	4.31, m	68.8	4.32, m
10	23.8	1.27, d (6.4)	23.8	1.28, d (6.5)
11	26.0	1.15, s	25.7	1.15, s
12	16.5	0.84, s	14.5	0.75, s
13	23.8	1.88, brs	23.8	1.87, t (1.2)
1′	104.9	4.41, d (7.8)		
2′	75.7	3.30, overlap		
3′	78.3	3.39, t (9.0)		
4′	71.5	3.29, overlap		
5′	78.2	3.24, overlap		
6′a	62.9	3.86, dd (11.8, 2.2)		
6′b		3.66, dd (11.8, 5.9)		

The strong NOESY cross-peaks of H-2 with H-6 and H-11 and H-6 with H-11 indicated that these three protons are co-facial (Figure 2B and Supporting Information File 1). The absolute configuration of C-6 was assigned as *S* by a well-established ECD empirical rule [22]. In brief, a systemic analysis of the ECD data of **13** with its diastereomers and simple derivatives showed that a positive or negative Cotton effect around 240–245 nm is indicative of the 6*R* or 6*S* configuration, respectively. From a positive Cotton effect at 245 nm of compound **1** (Figure 2C, left), the absolute configuration at C-6 was deduced as *S* (the stereochemical descriptor was flipped from *R* to *S* due to an *O*-glycosylation at C-2, see Figure 2C, right). The 9*R* configuration of **1** was determined by the modified Mosher’s method [23–25]. The hydrolysis product of **1** (**1a**) was esterified with the Mosher reagents and the analysis of the Δδ*_S_*_-_*_R_* values of all protons in **1a** indicated a 2*S* and 9*R* configuration (Figure 2D). Finally, the absolute configuration of the glucopyranose was assigned as ᴅ by comparing the retention time of its chiral derivative with those of authentic samples [16,26]. Therefore, the structure of compound **1** was elucidated as (2*S*,6*S*,7*E*,9*R*)-2,9-dihydroxy-4,7-megastigmadiene-3-one-2-*O*-β-ᴅ-glucopyranoside.

The 11 known compounds were identified as (6*S*,9*R*)-roseoside (**2**) [27], (*Z*)-4-[3'-(β-glucopyranosyloxy)butylidene]-3,5,5-trimethyl-2-cyclohexen-l-one (**3**) [28], betulalbuside A (**4**) [29], ethyl β-ᴅ-glucopyranoside (**5**) [30], *n*-butyl-α-ᴅ-fructofuranoside (**6**) [31], genistein 5-glucoside (**7**) [32], apigenin (**8**) [33], betulin (**9**) [3], betulinic acid (**10**) [34], betulinal (**11**) [35], and tormentic acid (**12**) [3] by comparison of their spectroscopic data with the reported data.

In the course of continuing search for cytotoxic, antineuroinflammatory, and neurotrophic secondary metabolites from *C. sinensis* [13–15,21], the isolates (**1–12**) were tested for these biological activities. The cytotoxicity was evaluated on the basis of the growth inhibitory effects of the isolated compounds **1–12** against four human tumor cell lines A549, SK-OV-3, SK-MEL-2, and BT549 using a sulforhodamine B (SRB) assay. Of the three lupane-type triterpenoids (**9–11**), betulin (**9**) and betulinic acid (**10**) showed potent cytotoxicity with IC_50_ values of 4.2–8.7 μM against all four tumor cell lines whereas betulinal (**11**) exhibited mild growth inhibitory effects against A549 and BT549 cell lines with IC_50_ values of 27.1 and 22.7 μM, respectively (Table 2). Betulinic acid (**10**) is a well-known anticancer agent inhibiting eukaryotic topoisomerase I [36] and its derivatives have been reported to display cytotoxic activity [13,37–40]. The acquired cytotoxicity data of compounds **9–11** are consistent with the previous studies. The other compounds **1–8** and **12** showed no activity (IC_50_ > 30 μM).

**Table 2:** Cytotoxicity of compounds **9–11** against four cultured human cancer cell lines in the SRB bioassay.

Compound	IC_50_ (μM)^a^			

	A549	SK-OV-3	SK-MEL-2	BT549

**9**	8.7	5.9	5.0	8.1
**10**	4.2	7.8	7.3	5.0
**11**	27.1	>30	>30	22.7
cisplatin^b^	1.0	2.2	1.8	3.5

^a^50% Inhibitory concentration; the concentration of compound that caused a 50% inhibition in cell growth. ^b^Positive control substance.

The potential antineuroinflammatory activity of the compounds **1–12** was tested by measuring the nitric oxide (NO) production levels in the LPS-stimulated murine microglia BV-2 cell line. Compound **11** (betulinal) showed a strong inhibitory effect on the NO production with an IC_50_ value of 5.7 μM without cytotoxicity (Table 3) which was 3.8-fold more potent than the positive control substance, ʟ-NMMA (IC_50_ 21.4 μM). Compounds **10** (betulinic acid) and **8** (apigenin) also exhibited significant activity with IC_50_ values of 4.3 and 16.6 μM, respectively. Tolstikov et al. reported that betulin (**9**) and its derivatives showed antiviral and antitumor effects [41]. Interestingly, betulinal (**11**) has an advantage in view of toxicity as compared to betulinic acid (**10**). A previous report suggested that apigenin (**8**) can lower the amyloid beta-induced microglial activation to inflammatory phenotype and this potential could help to lower the amyloid beta-induced neuroinflammation in CNS [42]. Additionally, an apigenin-mediated improvement in memory dysfunction in an animal model further elaborated the neuroprotective potential of apigenin itself [43]. Altogether, apigenin (**8**) could be a potential candidate for the treatment strategies against neurodegenerative disorders. However, the potency of compound **10** could be attributed to the low cell viability (54.9 ± 2.8%). The other phytochemicals displayed mild or no NO inhibitory effects (IC_50_ > 50 μM).

**Table 3:** Inhibitory effects of compounds **1–12** on the NO production in LPS-activated BV-2 cells.

Compound	IC_50_ (μM)^a^	cell viability (%)^b^

**1**	153.3	106.8 ± 4.3
**2**	150.0	116.4 ± 4.9
**3**	143.3	102.1 ± 3.0
**4**	156.7	104.2 ± 7.5
**5**	>500	85.5 ± 10.8
**6**	>500	108.8 ± 7.6
**7**	>500	90.6 ± 5.0
**8**	16.6	104.6 ± 11.7
**9**	120.1	71.8 ± 3.4
**10**	4.3	54.9 ± 2.8
**11**	5.7	125.6 ± 8.1
**12**	70.0	97.8 ± 10.8
ʟ-NMMA^c^	21.4	120.1 ± 11.7

^a^The IC_50_ value of each compound is defined as the concentration (μM) that caused 50% inhibition of the NO production in LPS-activated BV-2 cells. ^b^The cell viability following treatment with 20 μM of each compound was determined using the MTT assay and is expressed as a percentage (%). Data are expressed as the means ± SD of three independent experiments. ^c^Positive control substance.

Also, the potential neurotrophic effect of the isolated compounds **1**–**12** was evaluated by assessing their induction levels of nerve growth factor (NGF) secretion in C6 cells (Table 4). Among the tested compounds, only compound **8** (apigenin) exhibited a weak activity on the NGF release with a stimulation level of 127.8 ± 2.0% without displaying cell toxicity (100.9 ± 0.6%).

**Table 4:** Effects of compounds **1–12** on the NGF secretion in C6 cells.

Compound	NGF secretion^a^ (%)	cell viability^b^ (%)

**1**	105.8 ± 1.1	95.6 ± 5.5
**2**	86.8 ± 3.8	95.9 ± 1.9
**3**	95.4 ± 5.0	113.0 ± 10.6
**4**	91.4 ± 0.4	93.8 ± 1.4
**5**	101.5 ± 4.1	95.7 ± 9.5
**6**	95.6 ± 2.8	106.5 ± 11.0
**7**	115.3 ± 1.0	97.2 ± 10.4
**8**	127.8 ± 2.0	100.9 ± 0.6
**9**	111.6 ± 12.7	67.3 ± 1.3
**10**	101.5 ± 6.1	60.5 ± 4.1
**11**	100.1 ± 4.0	92.7 ± 0.2
**12**	105.6 ± 6.6	105.5 ± 0.1
6-shogaol^c^	149.5 ± 5.3	97.0 ± 0.2

^a^C6 cells were treated with 20 μM of each test compound. After 24 h, the content of NGF secreted in the C6-conditioned medium was measured by ELISA. The level of secreted NGF is expressed as the percentage of the untreated control (set as 100%). ^b^Cell viability after the treatment with 20 μM of each compound was determined by an MTT assay and is expressed as a percentage (%). Results are the means of three independent experiments, and the data are expressed as means ± SD. ^c^Positive control substance.

## Conclusion

A new megastigmane-type norsesquiterpenoid glycoside **1** was isolated along with 11 known compounds from the MeOH extract of *C. sinensis* twigs and their structures were characterized by intensive 1D and 2D NMR data analysis, ECD experiment, modified Mosher’s method, and LC–MS analysis. The 12 phytochemicals were evaluated for their anticancer, antineuroinflammatory, and neurotrophic effects and the active compounds **8–11** could be new drug candidates although further studies are needed. This phytochemical study on *C. sinensis* may exemplify, how novel secondary metabolites still remain undiscovered among the numerous well-known plant species.

## Experimental

**General experimental procedures**. Optical rotation data were recorded using a JASCO P-1020 polarimeter (JASCO, Easton, MD, USA). The NMR studies were accomplished employing a Bruker AVANCE III 700 NMR spectrometer (Bruker, Karlsruhe, Germany) and the resultant spectra were processed using MestReNova (Mnova, version 14.1.2-25024) with default weighting functions. HRFABMS data were acquired on a Waters SYNAPT G2 (Milford, MA, USA). The HPLC–DAD–MS data were measured using an Agilent 1260 Infinity HPLC system (Agilent, Santa Clara, CA, USA) with a Kinetex C18 5 μm column (250 mm length × 4.6 mm i.d.; Phenomenex, Torrance, CA, USA). Purification was achieved using a semi-preparative HPLC system equipped with a Gilson 306 pump (Middleton, WI, USA), a Shodex refractive index detector (New York, NY, USA), a Luna C_18_ 10 µm column (250 mm length × 10 mm i.d.; Phenomenex, Torrance, CA, USA), and an Apollo Silica 5 μm column (250 mm length × 10 mm i.d.; Apollo, Manchester, UK) at a flow rate of 2 mL/min. Low pressure liquid chromatography (LPLC) was performed with a LiChroprep Lobar-A Si 60 column (Merck, Darmstadt, Germany) and an FMI QSY-0 pump. Open columns packed with silica gel 60 (70–230 and 230–400 mesh; Merck), RP-18 silica gel (230–400 mesh; Merck, Darmstadt, Germany), or Diaion^®^ HP-20 resin (Sigma, St. Louis, MO) were implemented for crude fractionation and separation. Precoated silica gel F_254_ plates and RP-18 F_254s_ plates (Merck, Darmstadt, Germany) were utilized for thin-layer chromatography (TLC) and the spots were detected under UV light or by heating after spraying anisaldehyde–sulfuric acid.

**Plant material.** Twigs of *C. sinensis* were collected in Seoul, Republic of Korea in January 2012. A voucher specimen for the plant (SKKU-NPL 1206) was authenticated by Prof. Dr. Kang Ro Lee (Sungkyunkwan University) and stored at the herbarium of the School of Pharmacy, Sungkyunkwan University, Suwon, Republic of Korea.

**Extraction and isolation.** Extraction and solvent partitions were performed in the same manner as described in [13]. The hexanes-soluble fraction (3 g) was separated over a silica gel column (hexanes/EtOAc 3:1) to yield seven fractions (H1–H7). Fraction H3 (0.4 g) was chromatographed on an RP-C18 silica gel column (90% aqueous MeOH) to yield 12 subfractions (H3-1–H3-12), and compound **11** (5 mg) was obtained from subfraction H3-12 (50 mg) by semi-preparative normal-phase HPLC (hexanes/EtOAc 8:1). The CHCl_3_-soluble fraction (15 g) was subjected to passage over a silica gel open column (CHCl_3_/MeOH 50:1 → 1:1) to furnish nine fractions (C1–C9). Fraction C4 (1.5 g) was fractionated into 12 subfractions (C4-1–C4-12) using an RP-C_18_ silica gel open column eluting with 70% aqueous MeOH. The subfraction C4-3 (30 mg) was purified by an isolation strategy using semi-preparative reversed-phase HPLC (85% aqueous MeOH) to yield compound **8** (5 mg). The EtOAc-soluble layer (6 g) was applied to a Sephadex LH-20 column with a solvent system of 90% aqueous MeOH to yield five fractions E1–E5. The subfraction E1 (3.8 g) was subjected to a silica gel column chromatography (CHCl_3_/MeOH 10:1) to give 15 subfractions (E1-1–E1-15). The subfraction E1-4 (80 mg) was separated using a Lobar-A RP-18 (50% aqueous MeOH) followed by semi-preparative reversed-phase HPLC (60% aqueous MeCN) to give compound **12** (5 mg). The combined subfractions E1-11 and E1-12 (390 mg) were fractionated using a Lobar-A RP-18 (40% aqueous MeOH) and compound **5** (6 mg) was isolated by further purification using semi-preparative reversed-phase HPLC (15% aqueous MeOH). The subfraction E1-13 (120 mg) was chromatographed using a Lobar-A RP-18 (50% aqueous MeOH) followed by semi-preparative reversed-phase HPLC (50% aqueous MeOH) to yield compound **7** (7 mg). The fraction E3 (0.3 g) was separated into two subfractions (E3-1 and E3-2) by silica gel column chromatography (CHCl_3_/MeOH 30:1) and the subfraction E3-2 (30 mg) was further separated using semi-preparative reversed-phase HPLC (90% aqueous MeCN) to give compounds **9** (9 mg) and **10** (5 mg). The *n*-BuOH-soluble layer (30 g) was applied to an open column packed with Diaion^®^ HP-20 resin pre-equilibrated with H_2_O. The column was washed with 1 L of H_2_O to remove polar molecules and then washed with 1 L of MeOH to give 9 g of a relatively nonpolar fraction. This fraction was subjected to a silica gel column chromatography (CHCl_3_/MeOH/H_2_O 3:1:0.1) to give ten subfractions B1–B10. The subfraction B3 (60 mg) was further purified by semi-preparative reversed-phase HPLC (40% aqueous MeOH) to give compounds **3** (3 mg) and **6** (2 mg). The subfraction B5 (0.5 g) was separated into nine subfractions (B5-1–B5-9) using an RP-C18 silica gel open column eluting with 40% aqueous MeOH. The compounds **1** (12 mg), **2** (3 mg), and **4** (5 mg) were obtained from subfractions B5-6 (30 mg), B5-4 (50 mg), and B5-7 (30 mg), respectively, by semi-preparative reversed-phase HPLC (20% aqueous MeCN).

**Chaemeloside (1).** Colorless gum; [α]_D_^21^ +48 (*c* 0.05, MeOH); UV (MeOH) λ_max_, nm (log ε): 223 (4.3), 283 (sh, 0.8); ECD (MeOH) λ_max_, nm (Δε) 245 (15.2), 290 (−2.4), 325 (1.4); ^1^H (700 MHz) and ^13^C NMR (175 MHz) data, see Table 1; HRMS–FAB (positive-ion mode, *m*/*z*): [M + Na]^+^ calcd for C_19_H_30_O_8_Na^+^, 409.1833; found, 409.1830.

**Enzymatic hydrolysis of compound 1.** Compound **1** (2.0 mg) was hydrolyzed with cellulase (10 mg) in 1 mL of H_2_O at 37 °C for 24 h. CHCl_3_ (1 mL × 2) was used to extract the organic components from the reaction mixture. The CHCl_3_-soluble phase was dried in vacuo to yield the aglycone **1a** (1 mg). To the dried water-soluble phase were added pyridine (0.5 mL) and ʟ-cysteine methyl ester hydrochloride (0.5 mg), and the reaction mixture was stirred at 60 °C for 1 h. Then, *o*-tolyl isothiocyanate (0.1 mL) was added and the mixture stirred at 60 °C for another 1 h. The reaction mixture was subjected without purification to LC–MS analysis (0.7 mL/min; 25% aqueous CH_3_CN with 0.1% formic acid for 30 min). The authentic samples of ᴅ-glucopyranose and ʟ-glucopyranose were derivatized and analyzed by the same method as described above. The hydrolysate derivative of compound **1** was detected at 23.3 min for ᴅ-glucopyranose by the LC–MS analysis, which corresponded with that of the ᴅ-form of the authentic sugar (23.3 min for ᴅ-glucopyranose and 21.4 min for ʟ-glucopyranose).

**Aglycone (1a).** Colorless gum; ^1^H (700 MHz) and ^13^C NMR (175 MHz) data, see Table 1.

**Cytotoxicity assessment.** The cytotoxicity of the purified compounds was tested against the A549 (non-small cell lung adenocarcinoma), SK-OV-3 (ovary malignant ascites), SK-MEL-2 (skin melanoma), and BT549 (invasive ductal carcinoma) cells, utilizing the sulforhodamine B colorimetric (SRB) method [44]. Cisplatin (≥98%; Sigma-Aldrich) served as a positive control.

**Assessment of the NO generation and cell viability.** Analogous as described in [45]. The BV-2 cells, developed by Dr. V. Bocchini at the University of Perugia (Perugia, Italy), were used for this study [46–47]. The cells were seeded in a 96-well plate (4 × 10^4^ cells/well) and incubated in the presence or absence of various doses of the tested compounds. Lipopolysaccharide (LPS, 100 ng/mL) was added to all wells containing the pretreated cells except the one for control and grown for 1 d. The produced levels of nitrite (NO_2_), a soluble oxidized product of NO, was evaluated with 0.1% *N*-1-naphthylethylenediamine dihydrochloride and 1% sulfanilamide in 5% phosphoric acid, aka the Griess reagent. The supernatant (50 μL) was mixed with the Gries reagent (50 μL) and after 10 min the absorbance was gauged at 570 nm. For a positive control, the reported nitric oxide synthase (NOS) inhibitor ʟ-NMMA was employed. Graded sodium nitrite solutions were utilized to determine the nitrite concentrations. An MTT assay was used for the cell viability assay.

**Nerve growth factor and cell viability assays.** Analogous as described in [13]. The C6 glioma cells (Korean Cell Line Bank, Seoul, Republic of Korea) were used to assess the release of NGF into the culture medium. The test cells were seeded onto 24-well plates at a density of 1 × 10^5^ cells/well. After 24 h, the cells were treated with serum-free DMEM and incubated with the designated concentrations of the compounds for an additional 24 h. The medium supernatant was collected from the culture plates and the NGF levels were evaluated using an ELISA development kit from R & D system (Minneapolis, MN, USA). The cell viability was also assessed with a 3-[4,5-dimethylthiazol-2-yl]-2,5-diphenyltetrazolium bromide (MTT) assay in which the results were expressed as a percentage of the control group (untreated cells).

## Supporting Information

File 1 1D and 2D NMR, HRMS, and ECD spectra of compound **1**, ^1^H and ^13^C NMR spectra of **1a**, and ^1^H NMR spectra of **1s** and **1r**.
