# Supplementary material for: Chemical constituents of Chaenomeles sinensis twigs and their biological activity
Source: Beilstein J Org Chem. 2020 Dec 17;16:3078–85. doi: 10.3762/bjoc.16.257 (PMC7753107; doi:10.3762/bjoc.16.257)

BEILSTEIN JOURNAL OF ORGANIC CHEMISTRY

Supporting Information

for

Chemical constituents of *Chaenomeles sinensis* twigs and their biological activity

Joon Min Cha, Dong Hyun Kim, Lalita Subedi, Zahra Khan, Sang Un Choi, Sun Yeou Kim and Chung Sub Kim

Beilstein J. Org. Chem. **2020,** *16,* 3078–3085. doi:10.3762/bjoc.16.257

1D and 2D NMR, HRMS, and ECD spectra of compound 1, ^{1}H and ^{13}C NMR spectra of 1a, and ^{1}H NMR spectra of 1s and 1r

License and Terms: This is a supporting information file under the terms of the Creative Commons Attribution License (https://creativecommons.org/licenses/by/4.0). Please note that the reuse, redistribution and reproduction in particular requires that the author(s) and source are credited and that individual graphics may be subject to special legal provisions.
The license is subject to the *Beilstein Journal of Organic Chemistry* terms and conditions: (https://www.beilstein-journals.org/bjoc/terms)

Table of contents

Figure S1. The HRMS–FAB spectrum of **1** S2
Figure S2. The ^{1}H NMR spectrum of **1** in methanol-d_4 .... S3
Figure S3. The ^{13}C NMR spectrum of **1** in methanol-d_4 .... S4
Figure S4. The COSY spectrum of **1** in methanol-d_4 .... S5
Figure S5. The HSQC spectrum of **1** in methanol-d_4 .... S6
Figure S6. The HMBC spectrum of **1** in methanol-d_4 .... S7
Figure S7. The NOESY spectrum of **1** in methanol-d_4 .... S8
Figure S8. The ECD spectrum of **1** in methanol S9
Figure S9. The ^{1}H NMR spectrum of **1a** in methanol-d_4 .... S10
Figure S10. The ^{13}C NMR spectrum of **1a** in methanol-d_4 .... S11
Figure S11. The ^{1}H NMR spectrum of **1s** in pyridine-d_5 .... S12
Figure S12. The ^{1}H NMR spectrum of **1r** in pyridine-d_5 .... S13
Table S1. ^{1}H [ppm, mult., (*J* in Hz)] of two Mosher esters **1s** and **1r** and the $\Delta\delta_{S\text{-}R}$ values in pyridine-d_5 .... S14
Figure S13. The ^{1}H NMR spectrum of **2** in methanol-d_4 .... S15
Figure S14. The ^{1}H NMR spectrum of **3** in methanol-d_4 .... S16
Figure S15. The ^{13}C NMR spectrum of **3** in methanol-d_4 .... S17
Figure S16. The ^{1}H NMR spectrum of **4** in methanol-d_4 .... S18
Figure S17. The ^{13}C NMR spectrum of **4** in methanol-d_4 .... S19
Figure S18. The ^{1}H NMR spectrum of **5** in methanol-d_4 .... S20
Figure S19. The ^{13}C NMR spectrum of **5** in methanol-d_4 .... S21
Figure S20. The ^{1}H NMR spectrum of **6** in methanol-d_4 .... S22
Figure S21. The ^{13}C NMR spectrum of **6** in methanol-d_4 .... S23
Figure S22. The ^{1}H NMR spectrum of **7** in methanol-d_4 .... S24
Figure S23. The ^{13}C NMR spectrum of **7** in methanol-d_4 .... S25
Figure S24. The ^{1}H NMR spectrum of **8** in methanol-d_4 + chloroform-*d* S26
Figure S25. The ^{13}C NMR spectrum of **8** in methanol-d_4 + chloroform-*d* S27
Figure S26. The ^{1}H NMR spectrum of **9** in methanol-d_4 .... S28
Figure S27. The ^{13}C NMR spectrum of **9** in methanol-d_4 .... S29
Figure S28. The ^{1}H NMR spectrum of **10** in methanol-d_4 + chloroform-*d* S30
Figure S29. The ^{13}C NMR spectrum of **10** in methanol-d_4 + chloroform-*d* S31
Figure S30. The ^{1}H NMR spectrum of **11** in chloroform-*d* S32
Figure S31. The ^{1}H NMR spectrum of **12** in pyridine-d_5 .... S33
Figure S32. The ^{13}C NMR spectrum of **12** in pyridine-d_5 .... S34

Figure S1. The HRMS–FAB spectrum of **1**

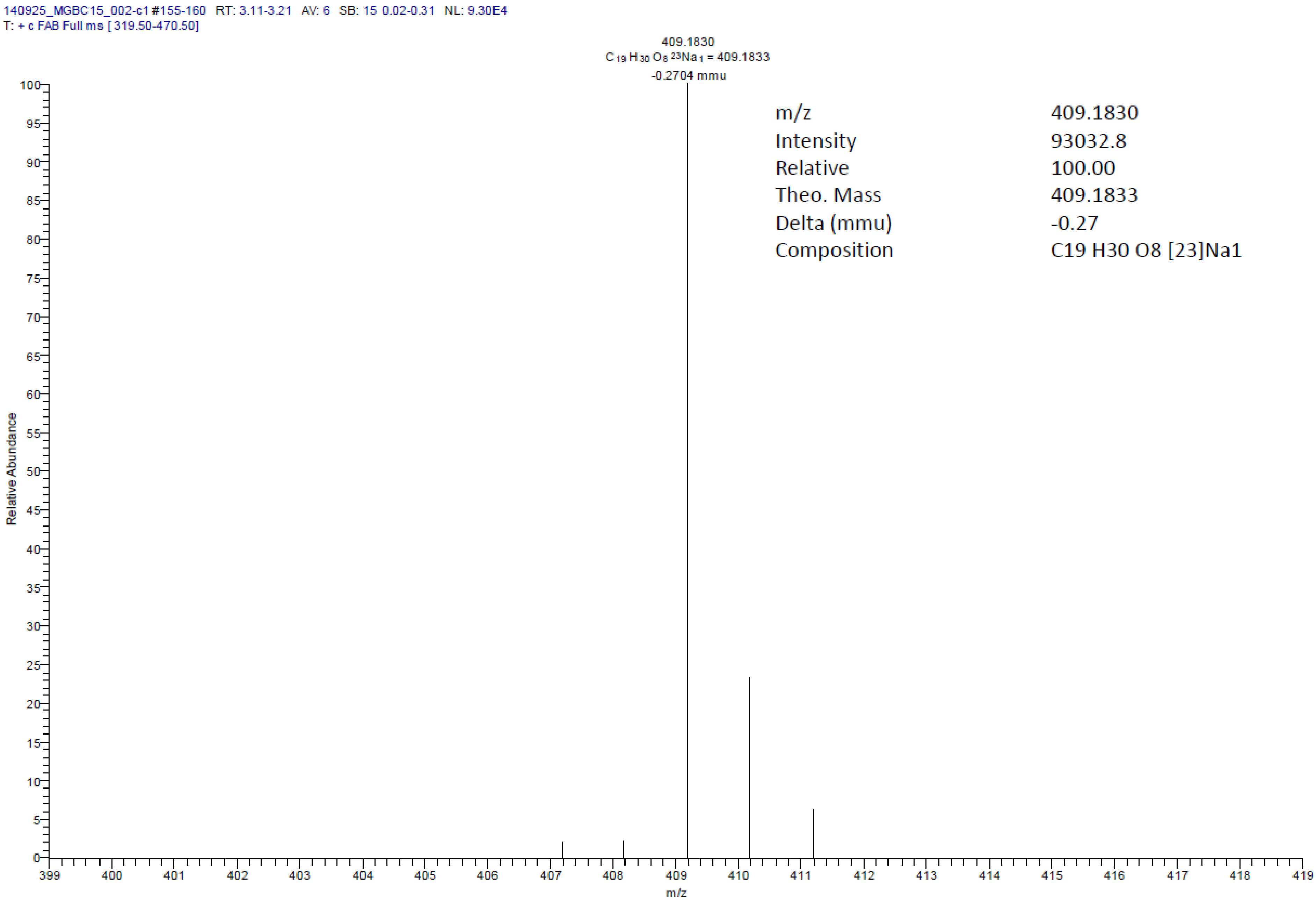

Figure S2. The ^{1}H NMR spectrum of **1** in methanol-d_4

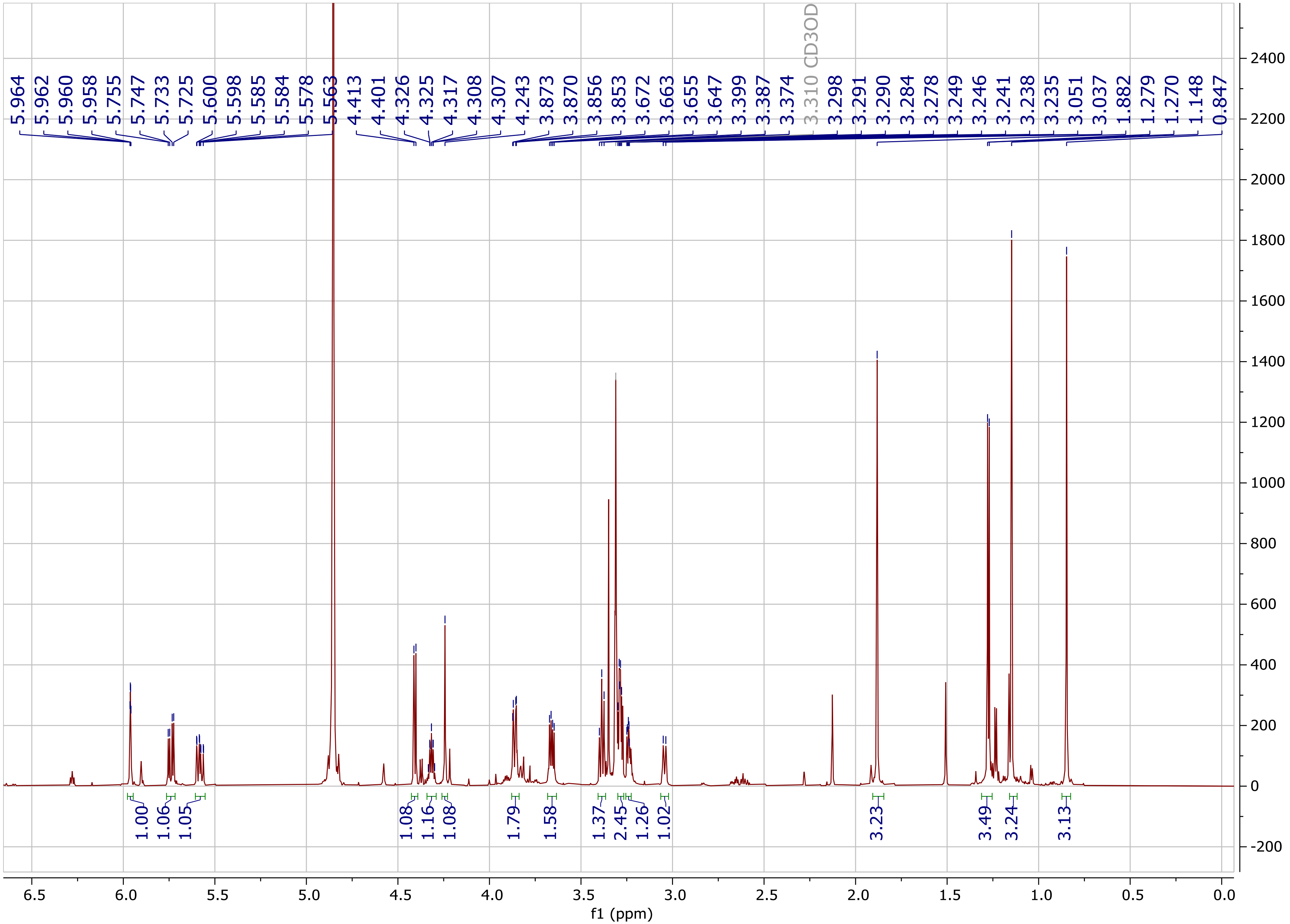

Figure S3. The ^{13}C NMR spectrum of **1** in methanol-d_4

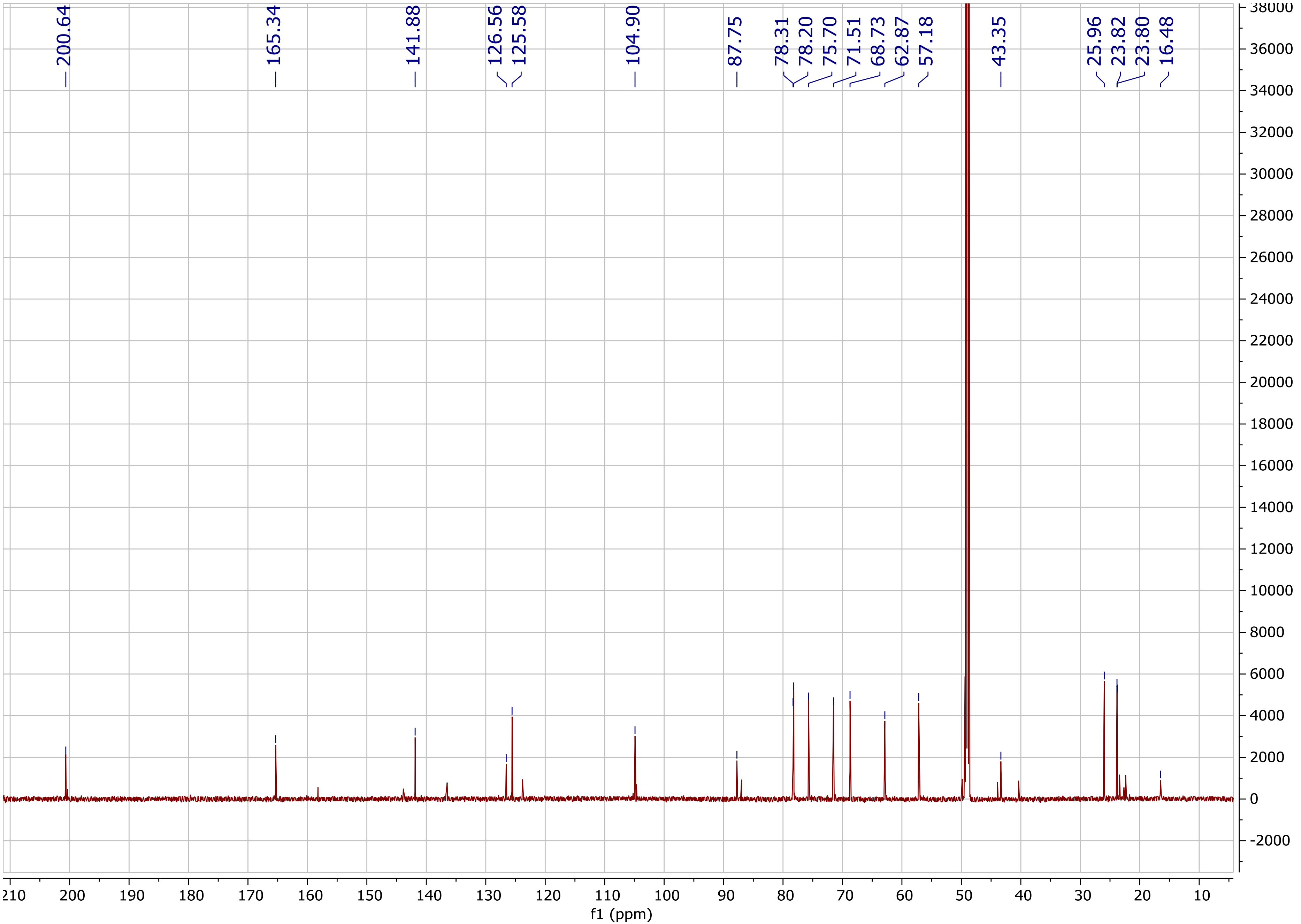

Figure S4. The COSY spectrum of **1** in methanol-d_4

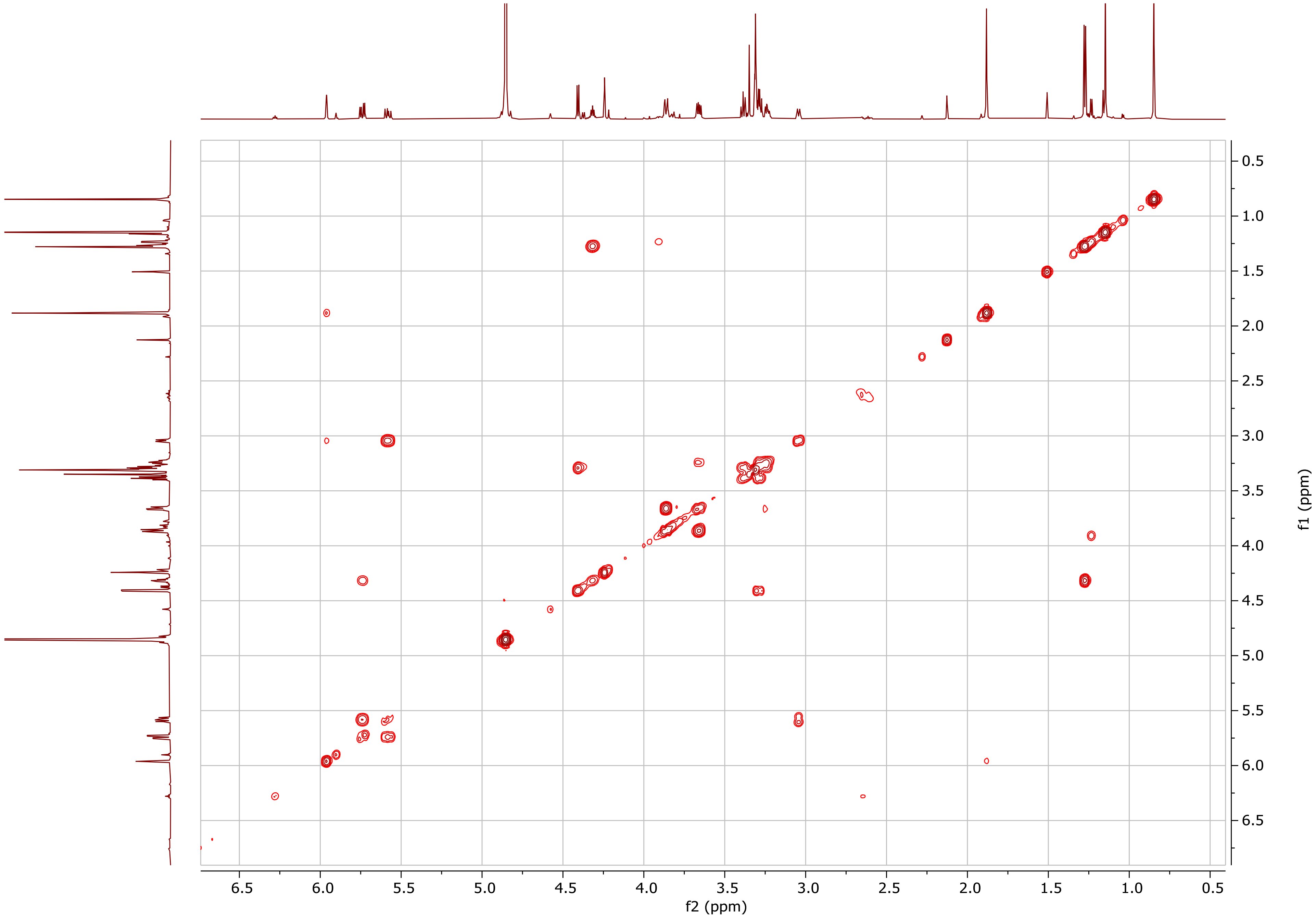

Figure S5. The HSQC spectrum of **1** in methanol-d_4

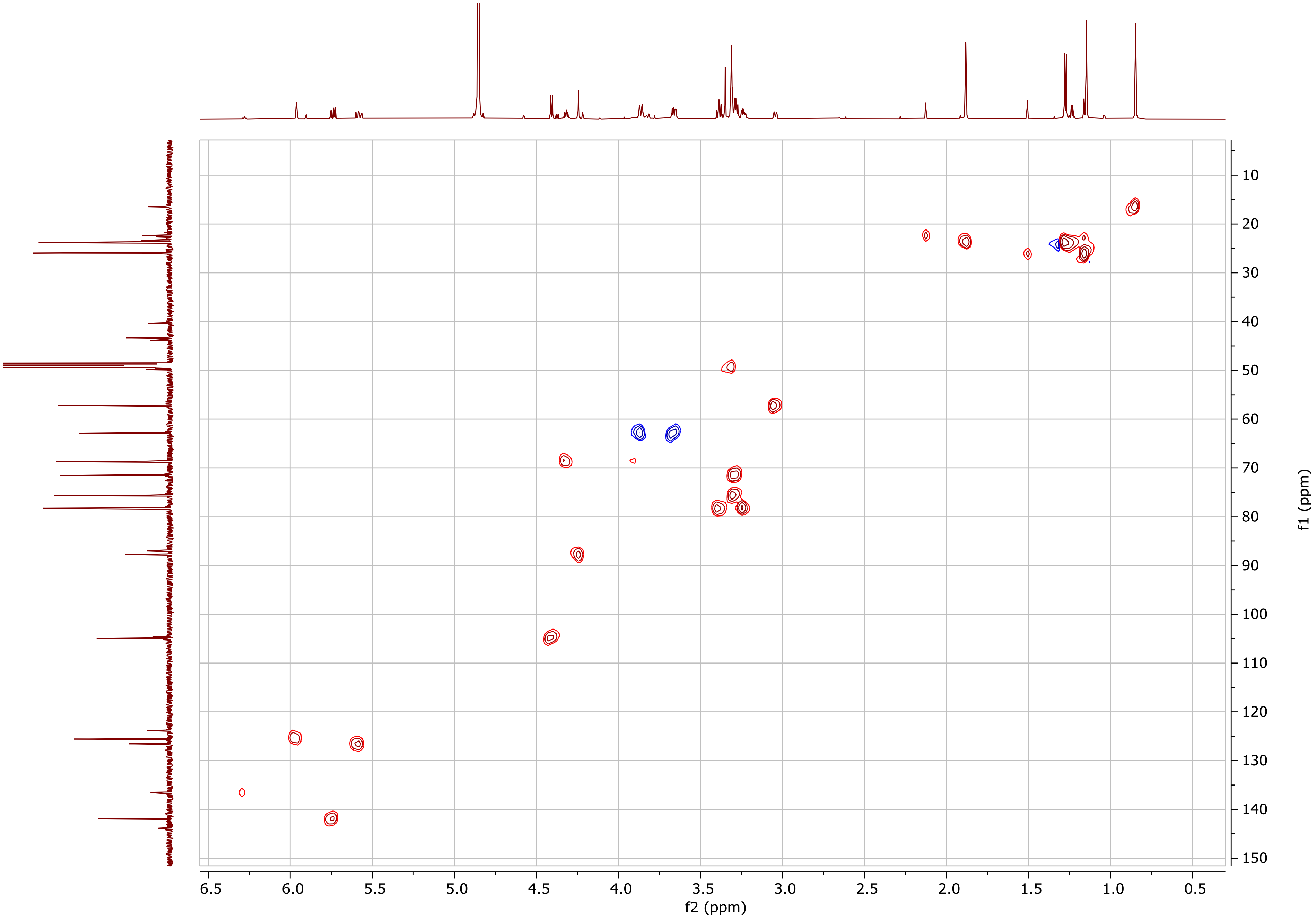

Figure S6. The HMBC spectrum of **1** in methanol-d_4

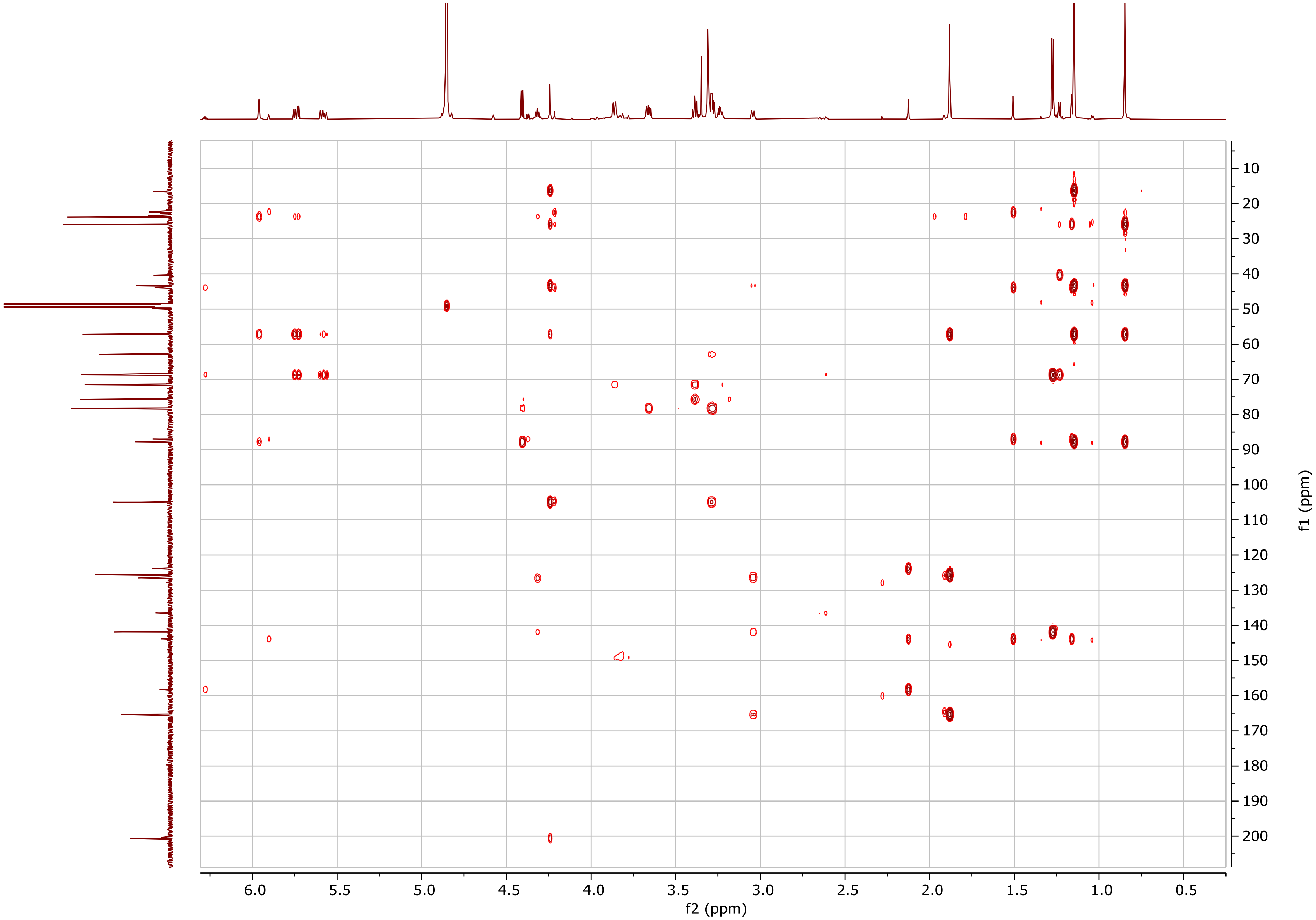

Figure S7. The NOESY spectrum of **1** in methanol-d_4

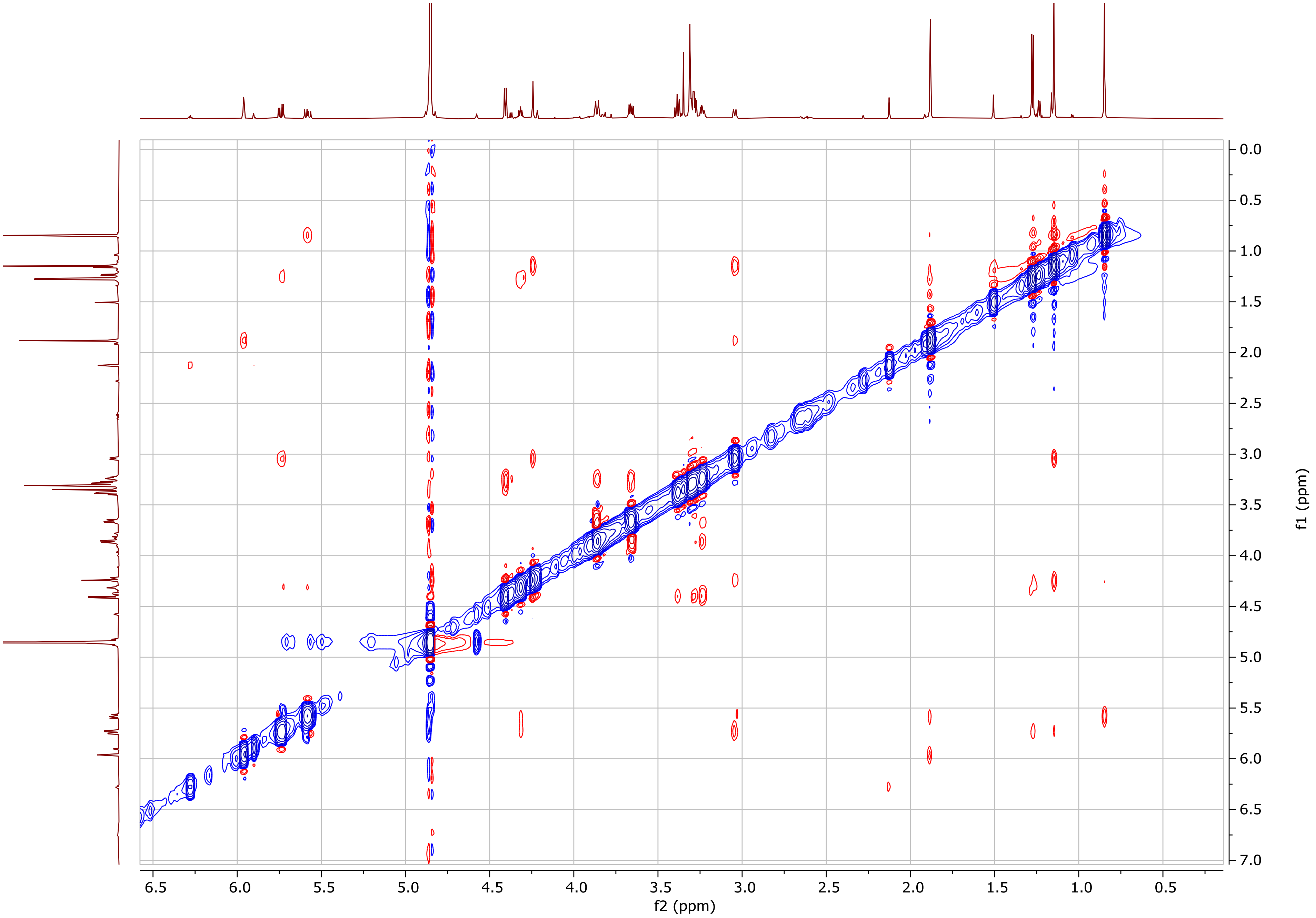

Figure S8. The ECD spectrum of **1** in methanol

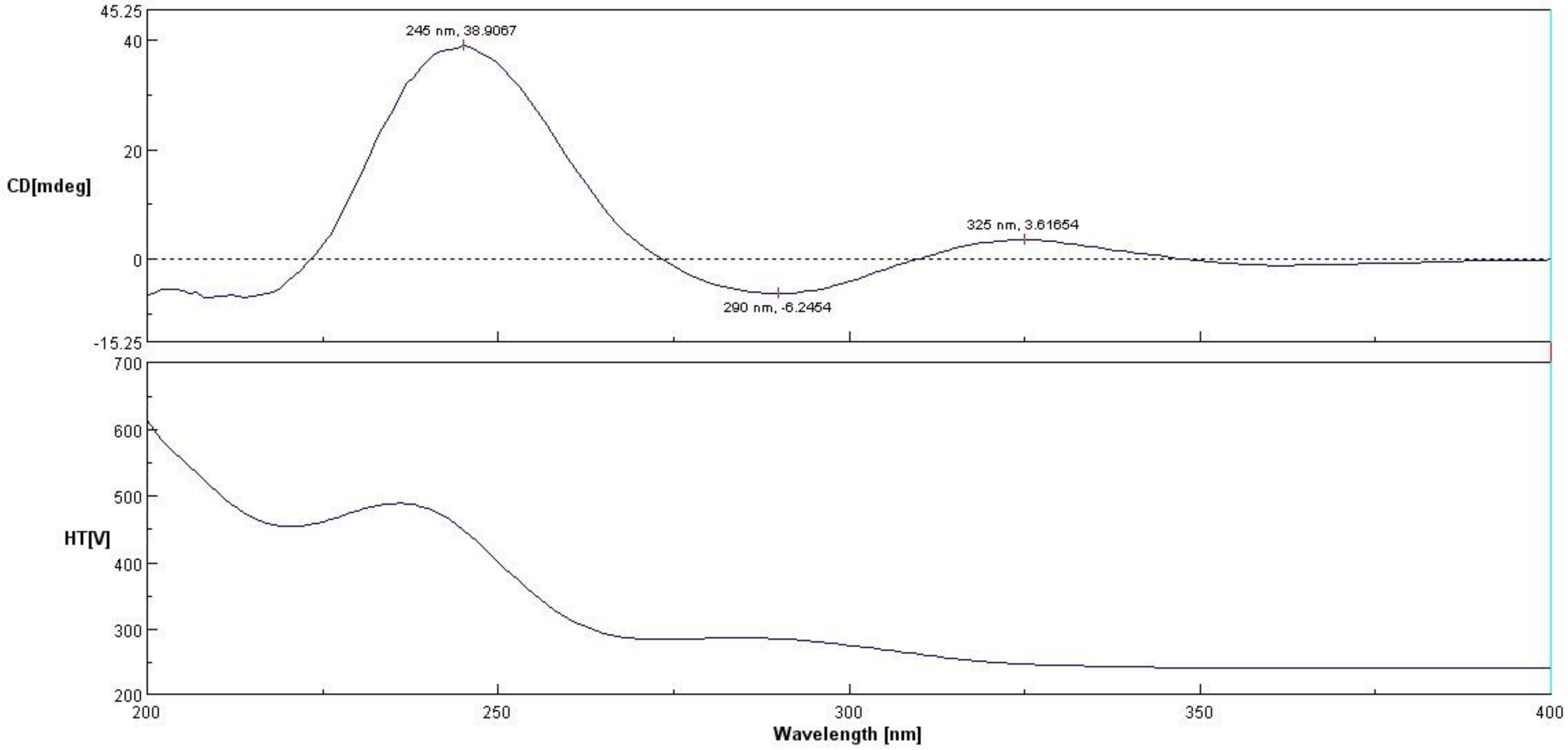

Figure S9. The ^{1}H NMR spectrum of **1a** in methanol-d_4

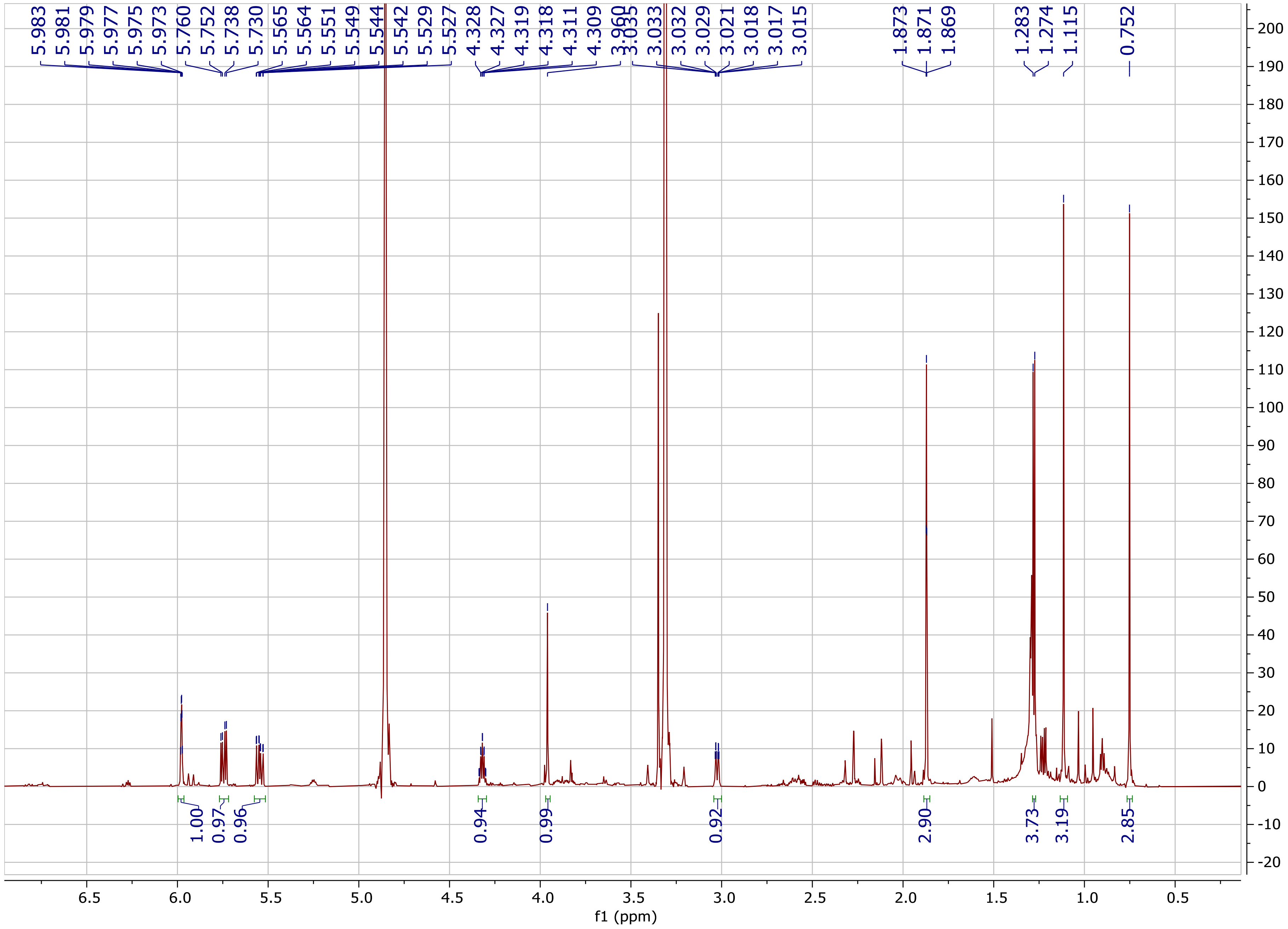

Figure S10. The ^{13}C NMR spectrum of **1a** in methanol-d_4

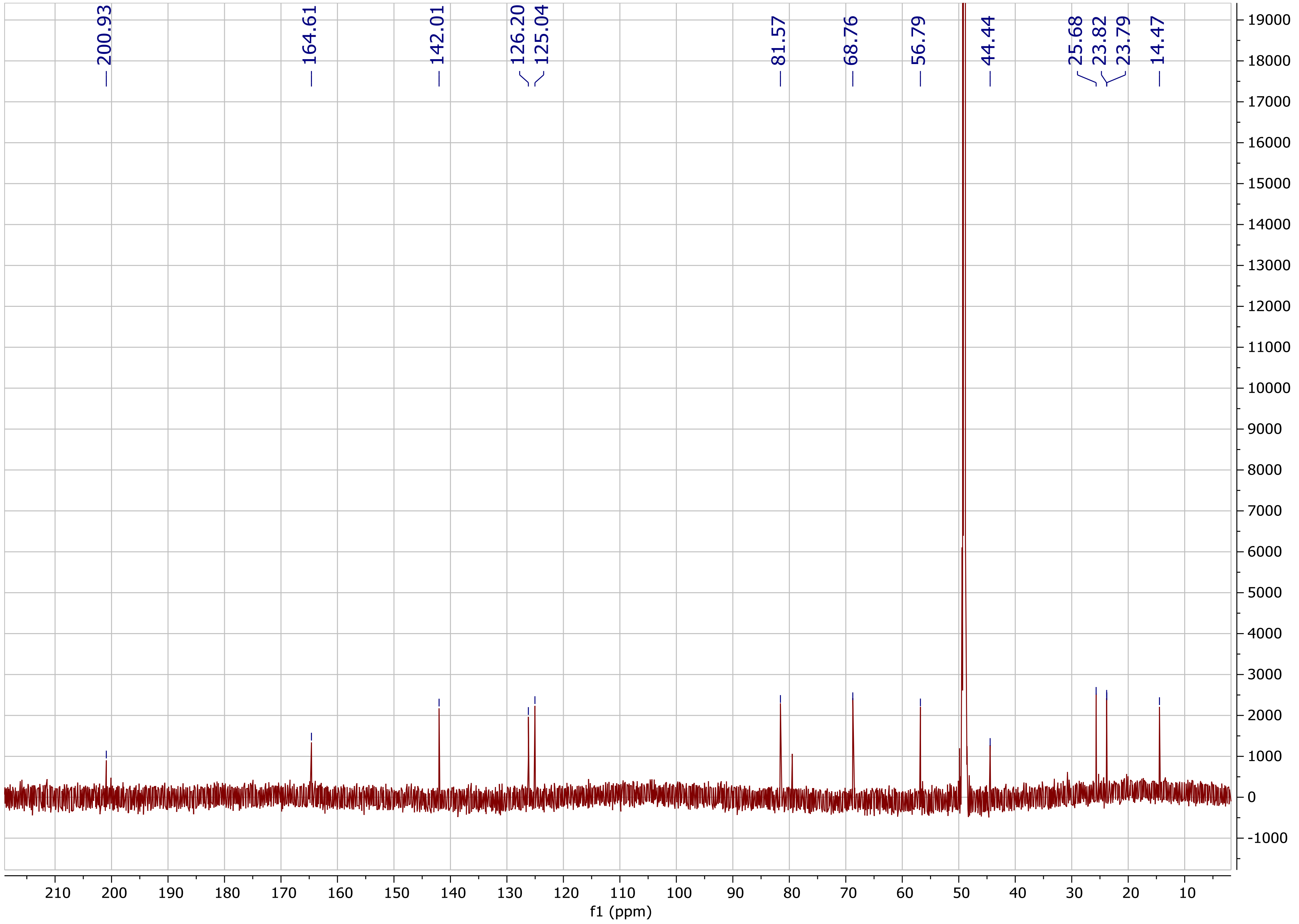

Figure S11. The ^{1}H NMR spectrum of **1s** in pyridine-d_5

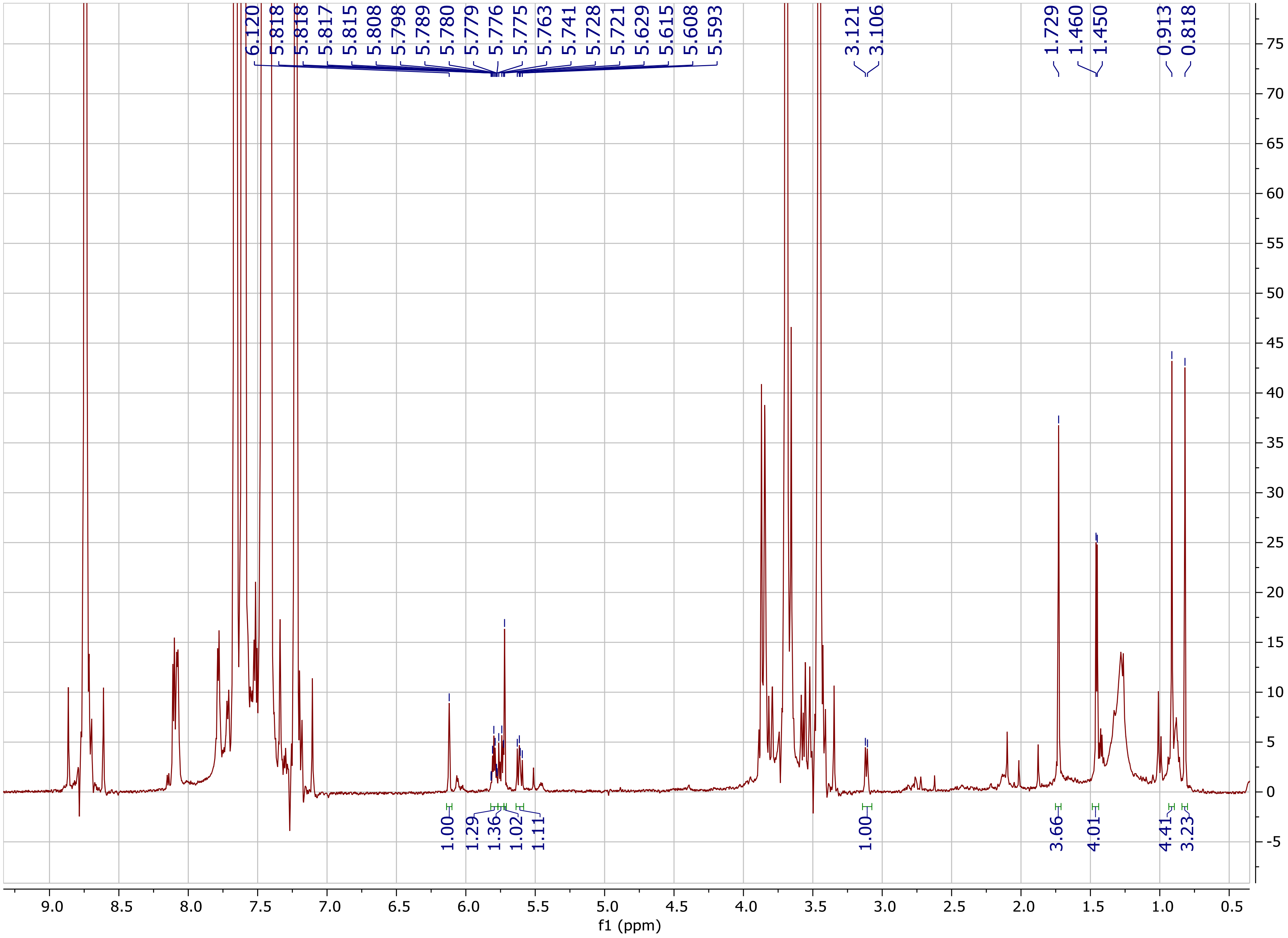

Figure S12. The ^{1}H NMR spectrum of **1r** in pyridine-d_5

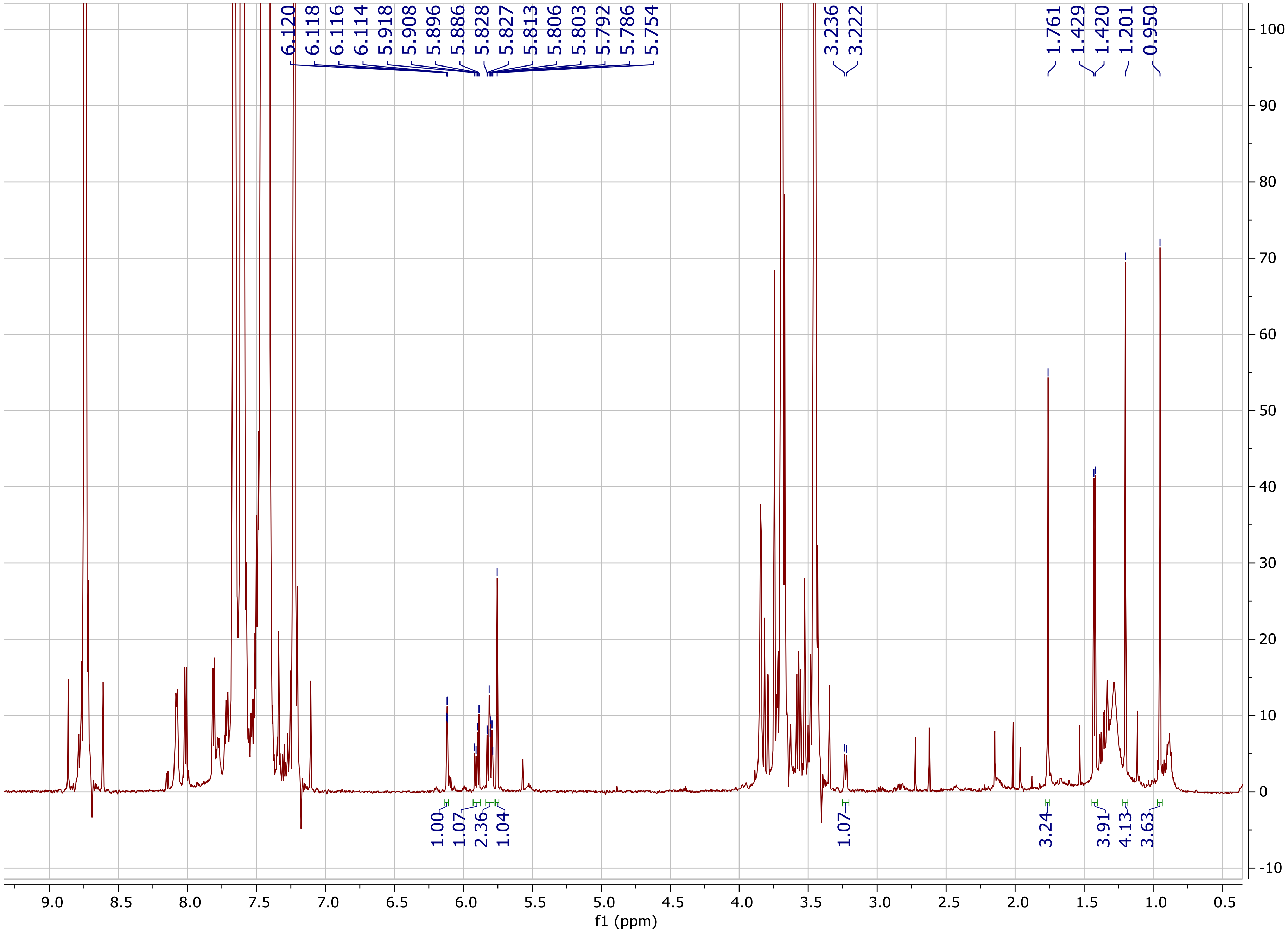

Table S1. ^{1}H [ppm, mult., (*J* in Hz)] of two Mosher esters **1s** and **1r** and the $\Delta\delta_{S\text{-}R}$ values in pyridine-d_5

Position	**1s**	**1r**	$\Delta\delta_{S\text{-}R}$
2	5.721, s	5.754, s	–0.033
4	6.120, m	6.116, m	+0.004
6	3.114, brd (10.5)	3.228, brd (10.0)	–0.114
7	5.611, dd (15.1, 10.3)	5.805, overlap	–0.194
8	5.747, overlap	5.902, dd (15.2, 7.3)	–0.155
9	5.798, m	5.813, m	–0.015
10	1.455, d (6.4)	1.425, d (6.4)	+0.030
11	0.913, s	1.201, s	–0.288
12	0.818, s	0.950, s	–0.132
13	1.729, s	1.761, s	–0.032

Figure S13. The ^{1}H NMR spectrum of **2** in methanol-d_4

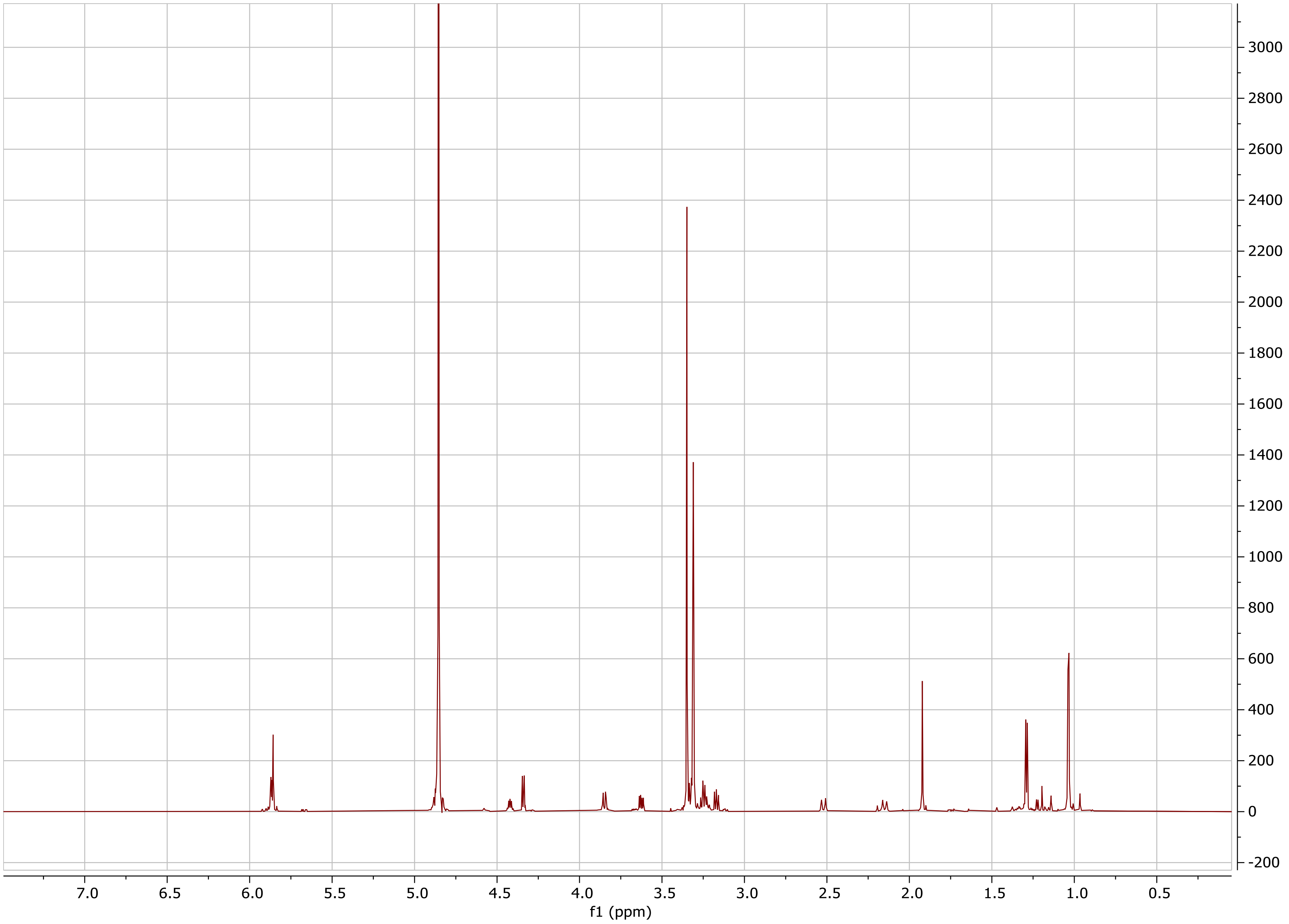

Figure S14. The ^{1}H NMR spectrum of **3** in methanol-d_4

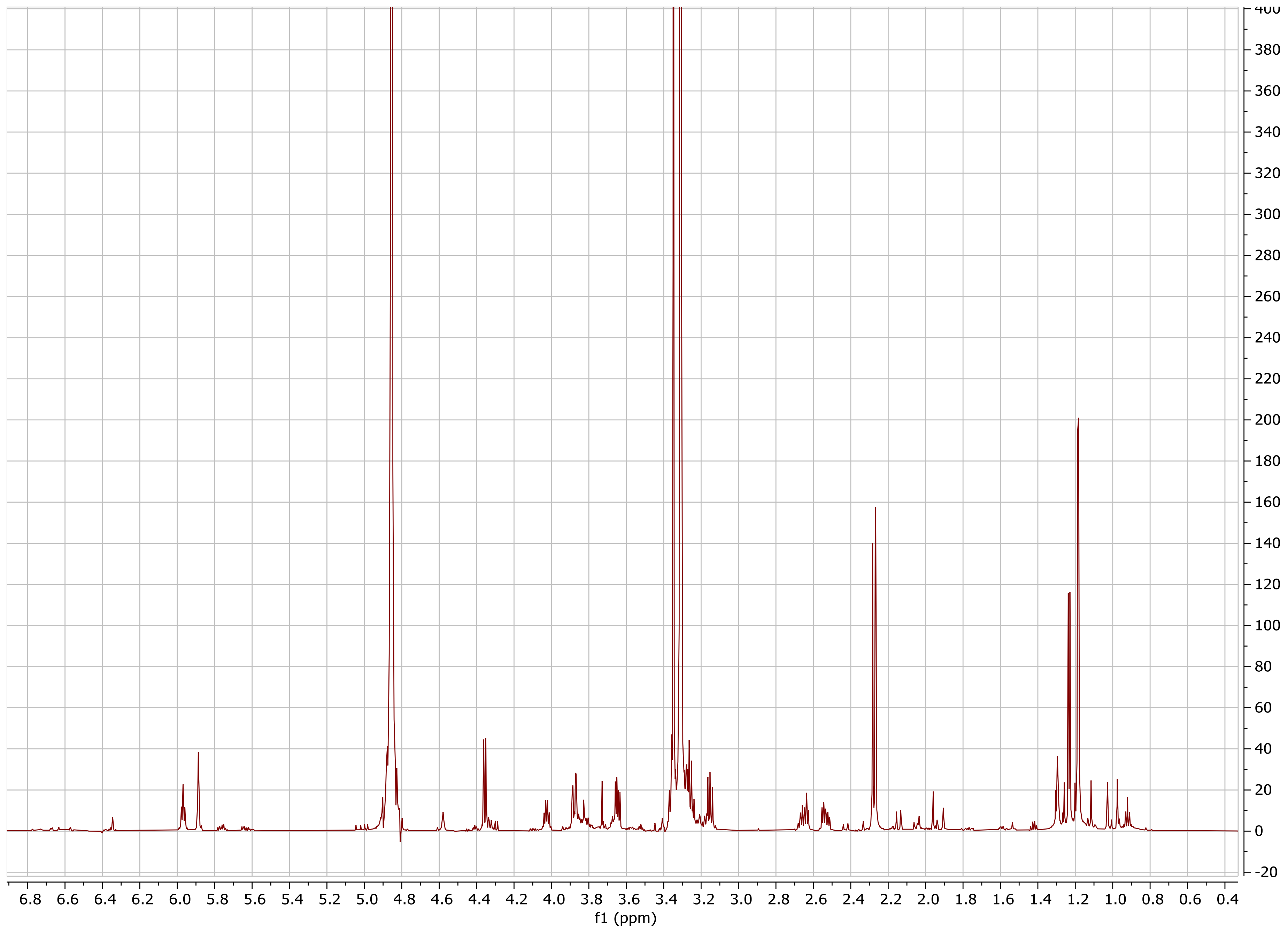

Figure S15. The ^{13}C NMR spectrum of **3** in methanol-d_4

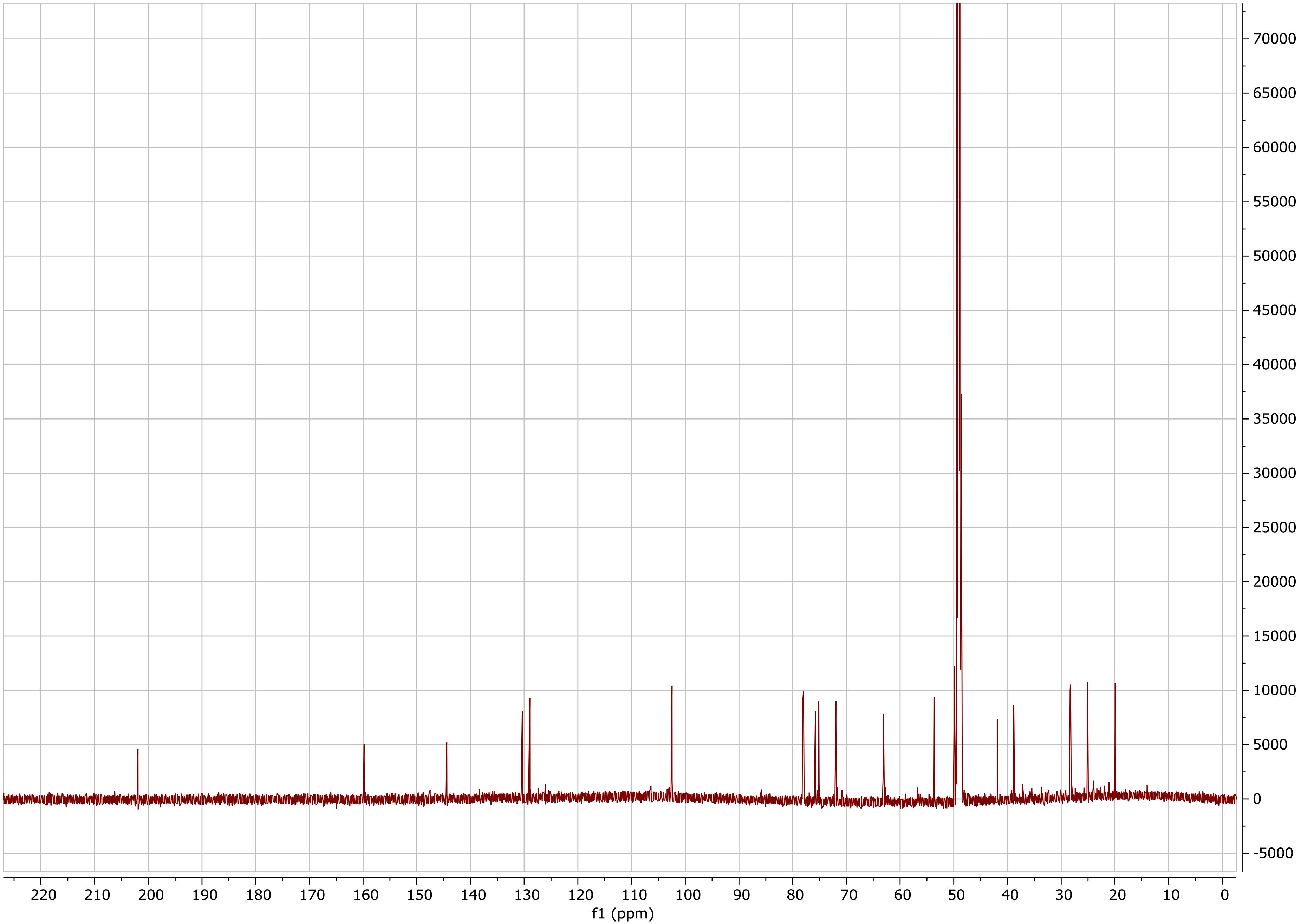

Figure S16. The ^{1}H NMR spectrum of **4** in methanol-d_4

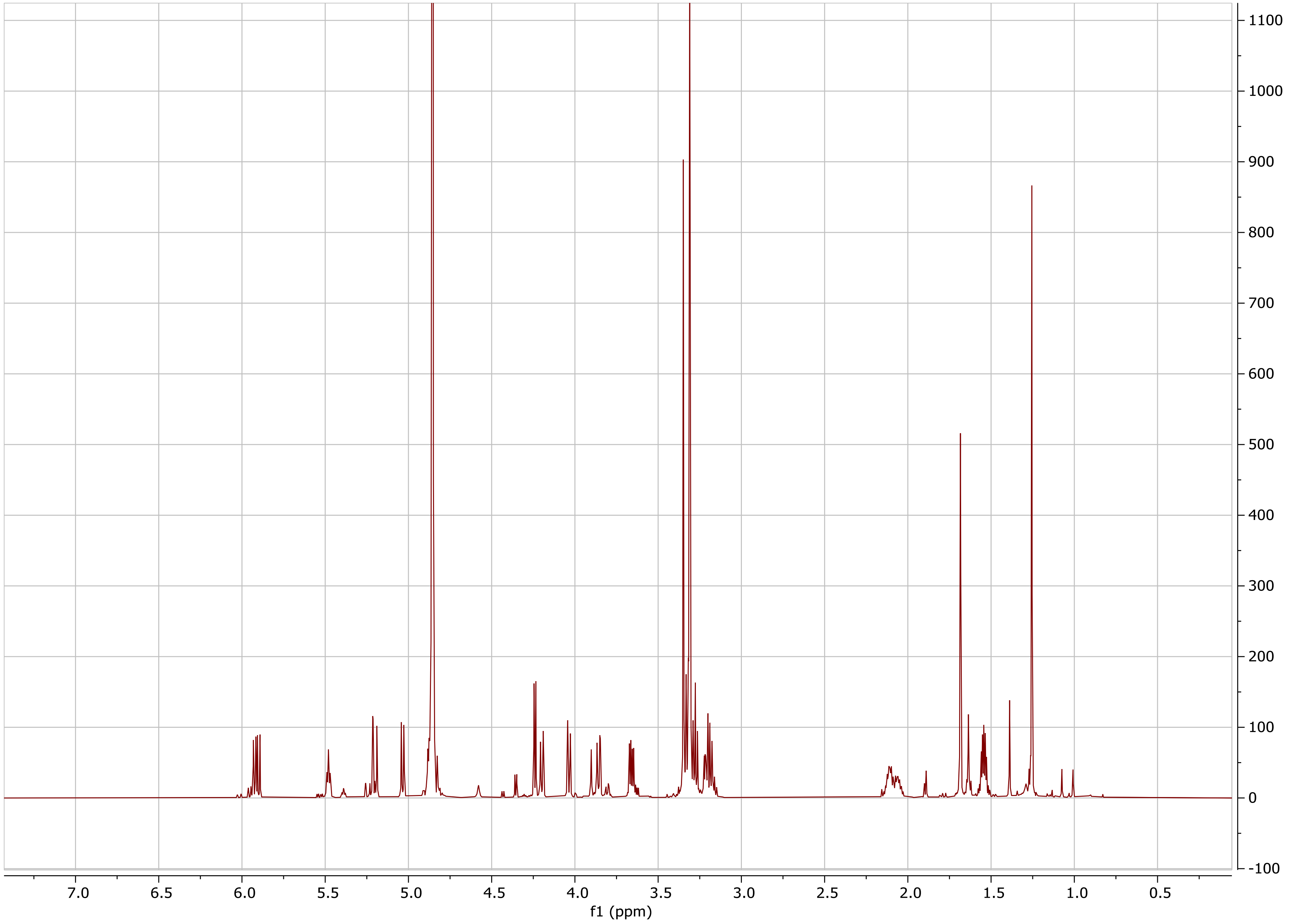

Figure S17. The ^{13}C NMR spectrum of **4** in methanol-d_4

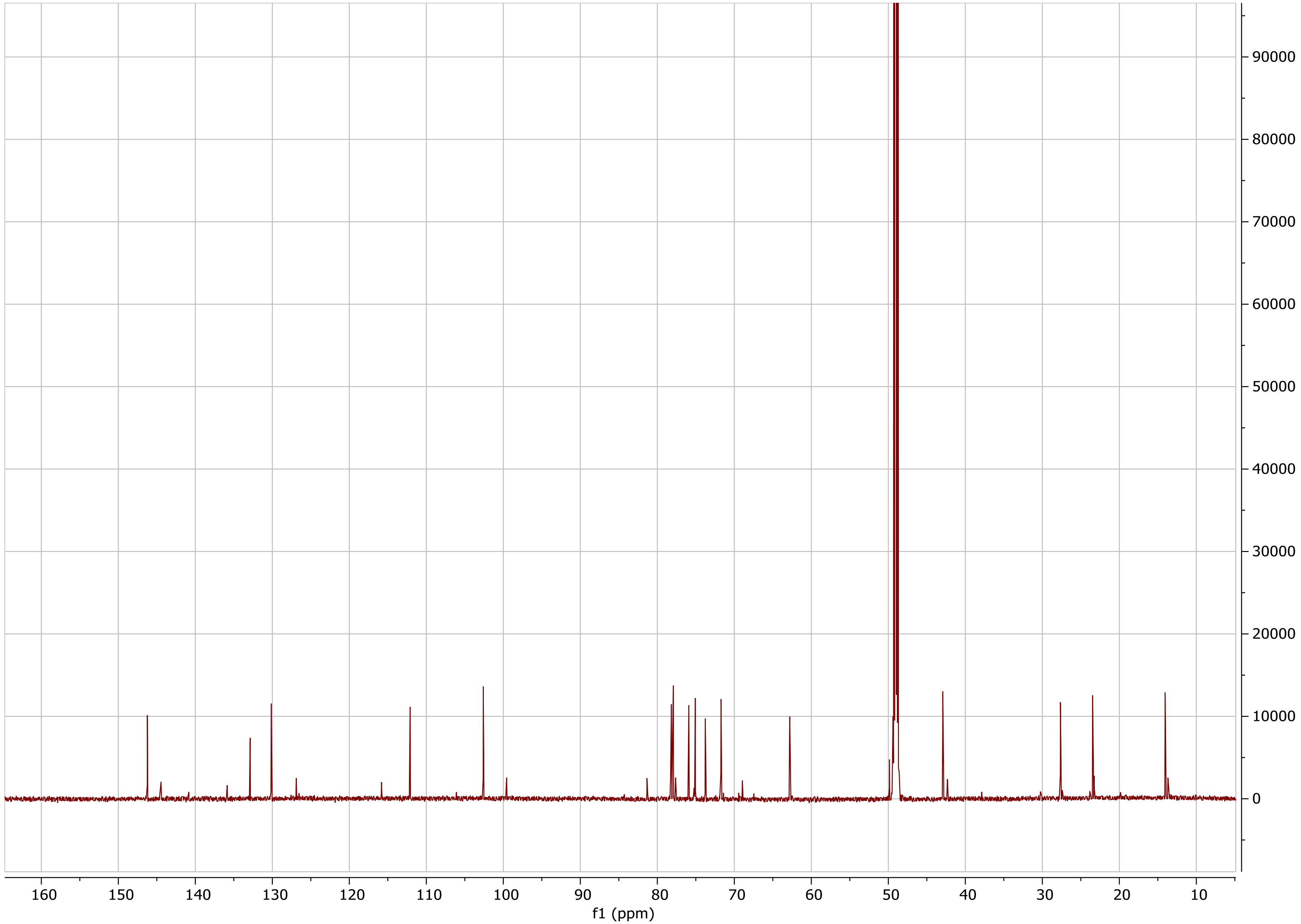

Figure S18. The ^{1}H NMR spectrum of **5** in methanol-d_4

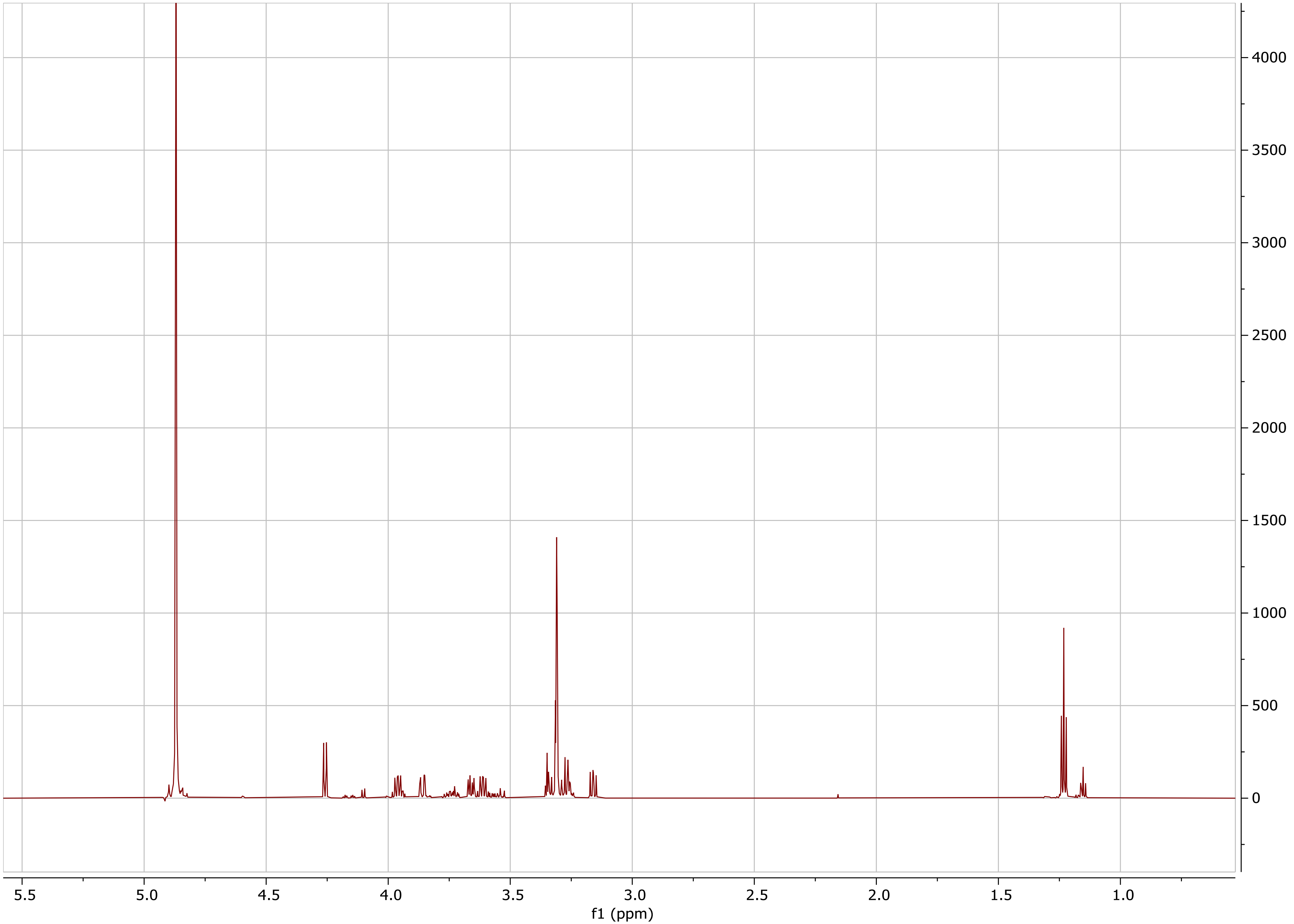

Figure S19. The ^{13}C NMR spectrum of **5** in methanol-d_4

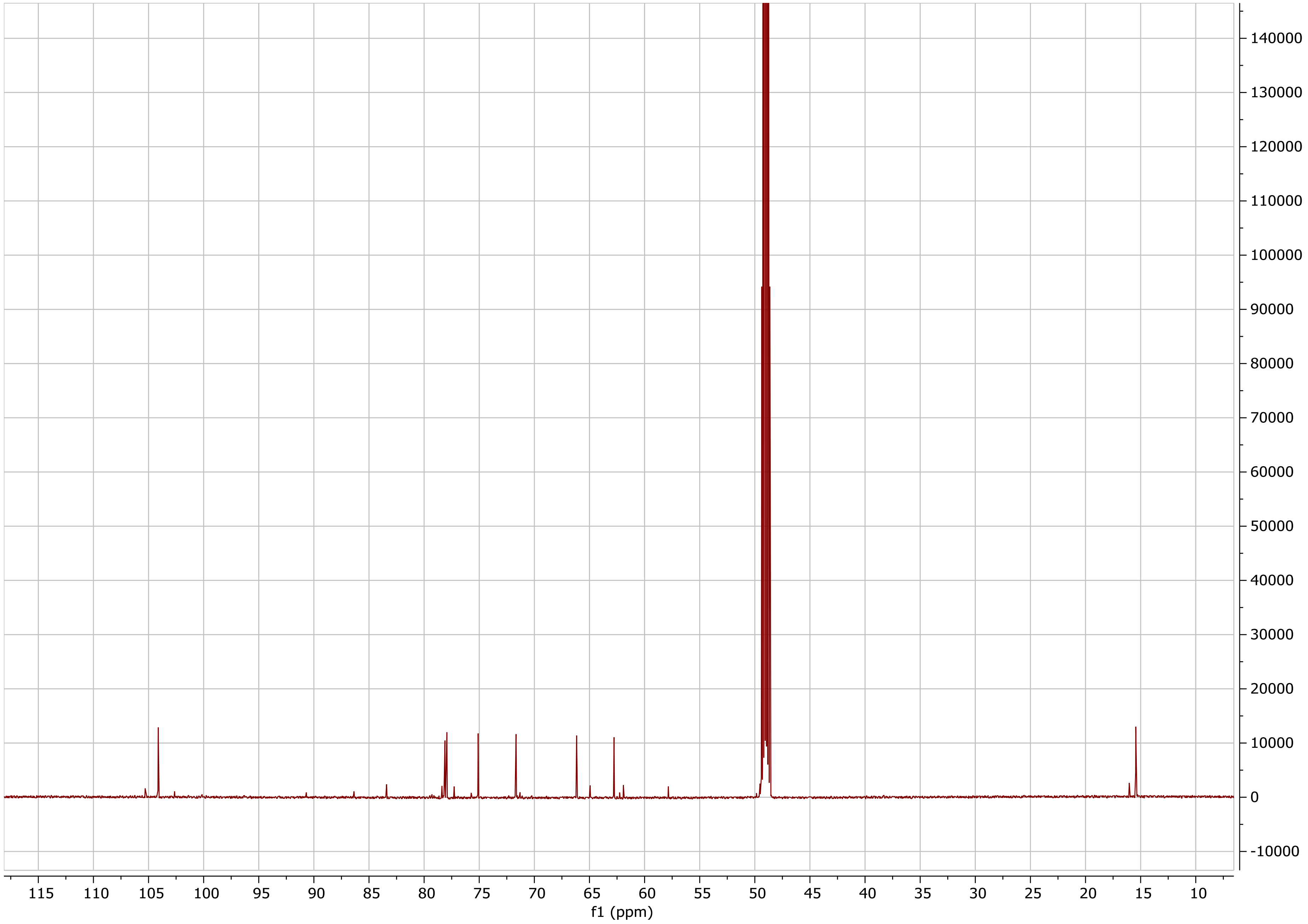

Figure S20. The ^{1}H NMR spectrum of **6** in methanol-d_4

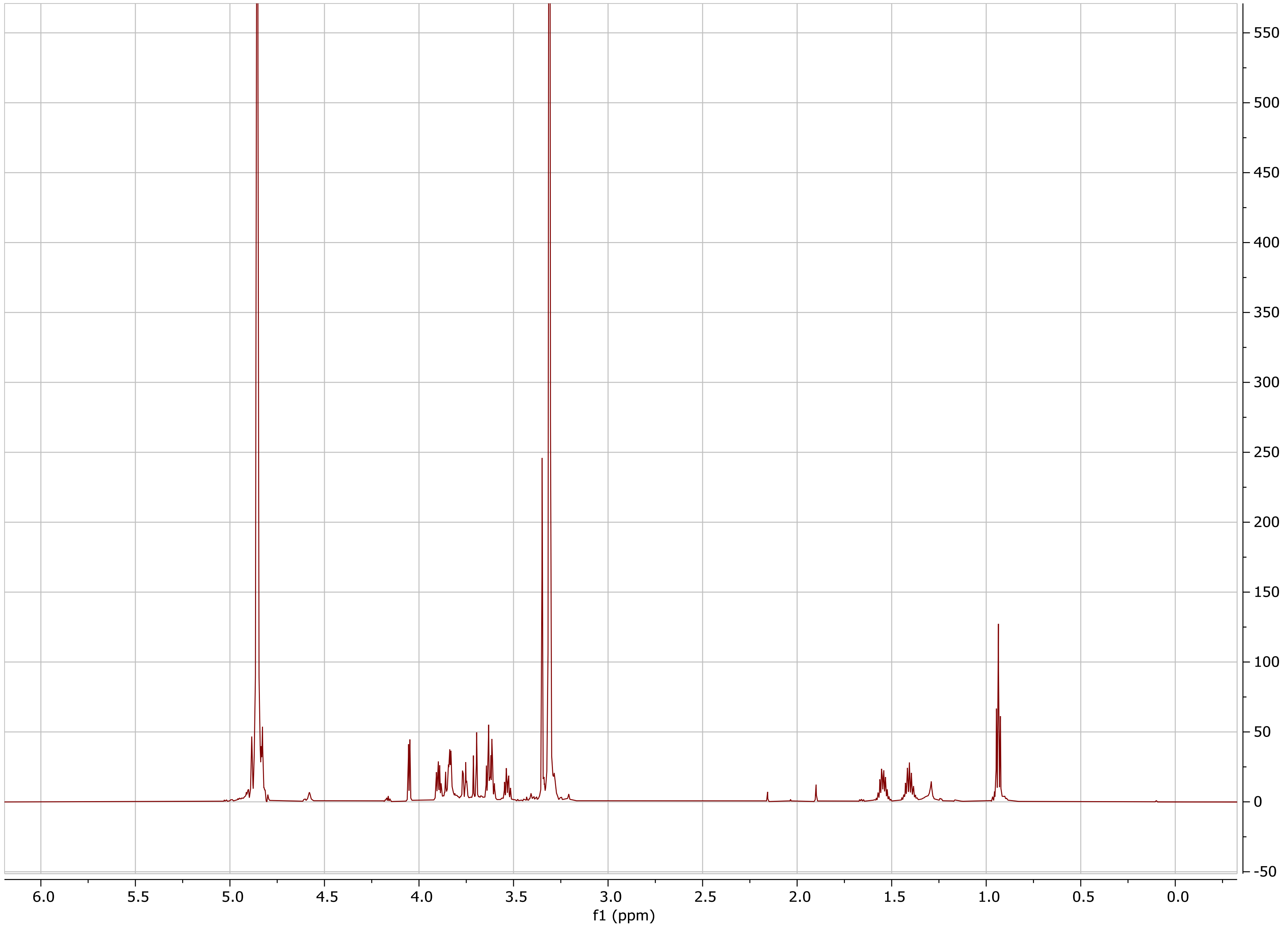

Figure S21. The ^{13}C NMR spectrum of **6** in methanol-d_4

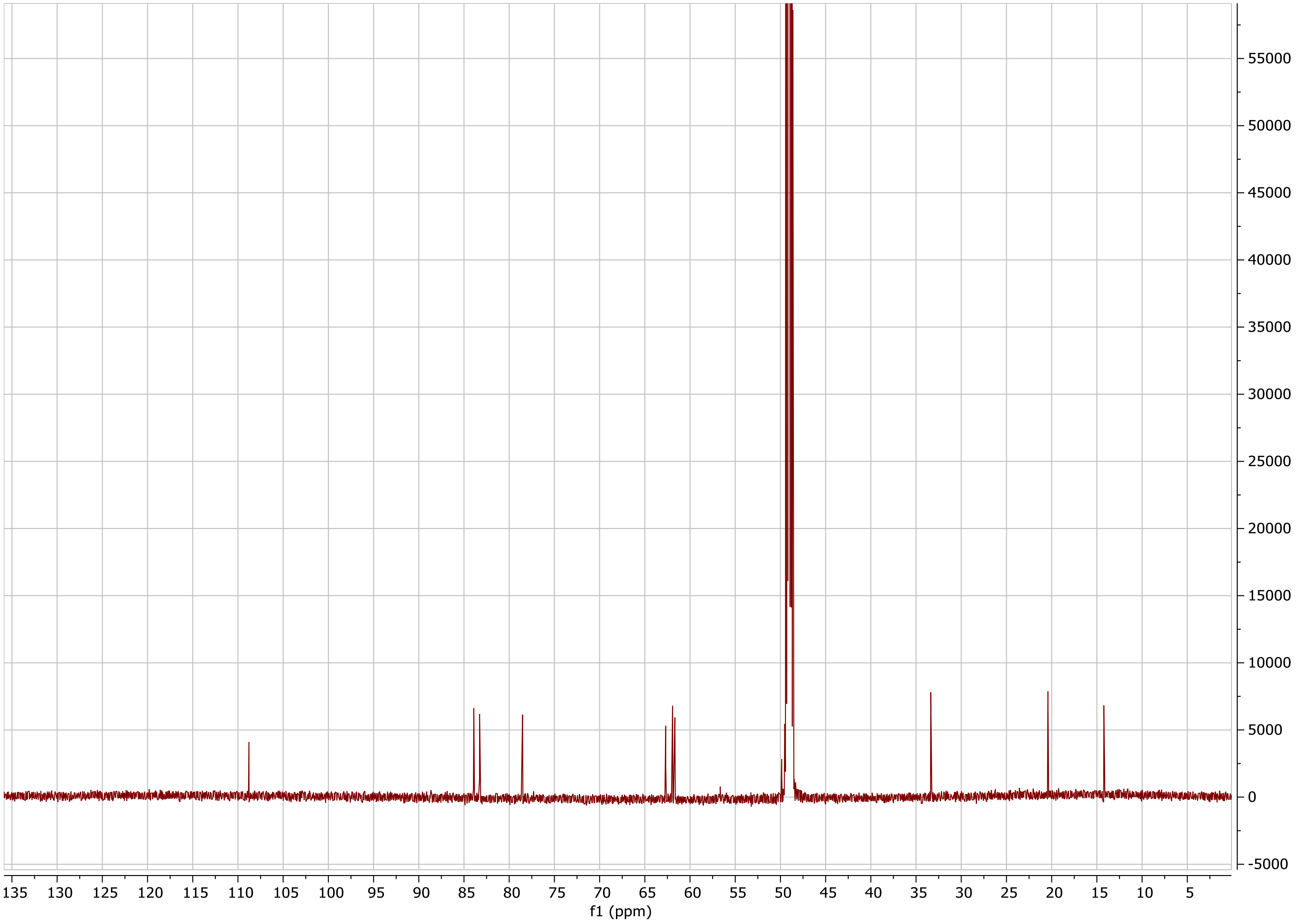

Figure S22. The ^{1}H NMR spectrum of **7** in methanol-d_4

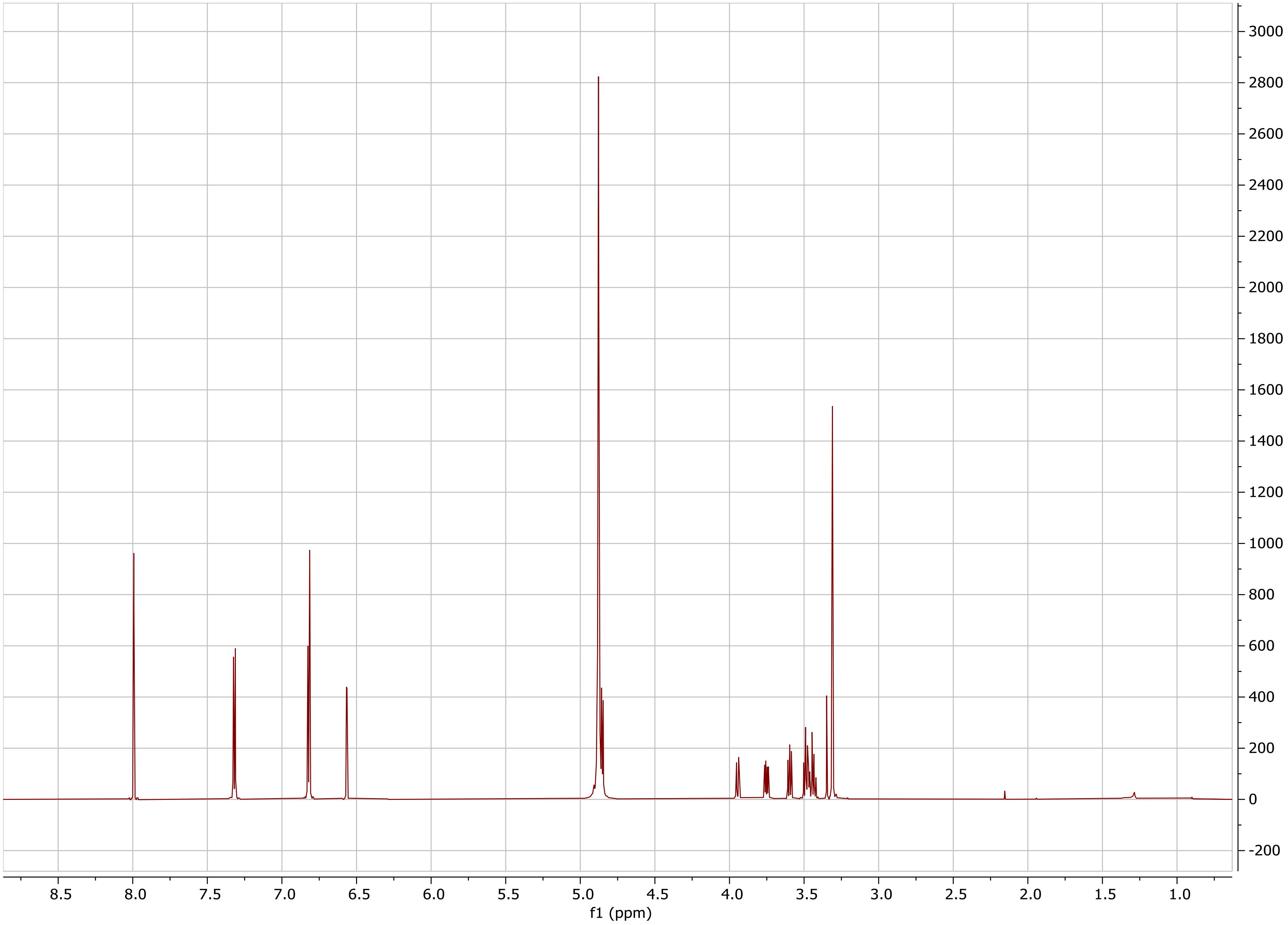

Figure S23. The ^{13}C NMR spectrum of **7** in methanol-d_4

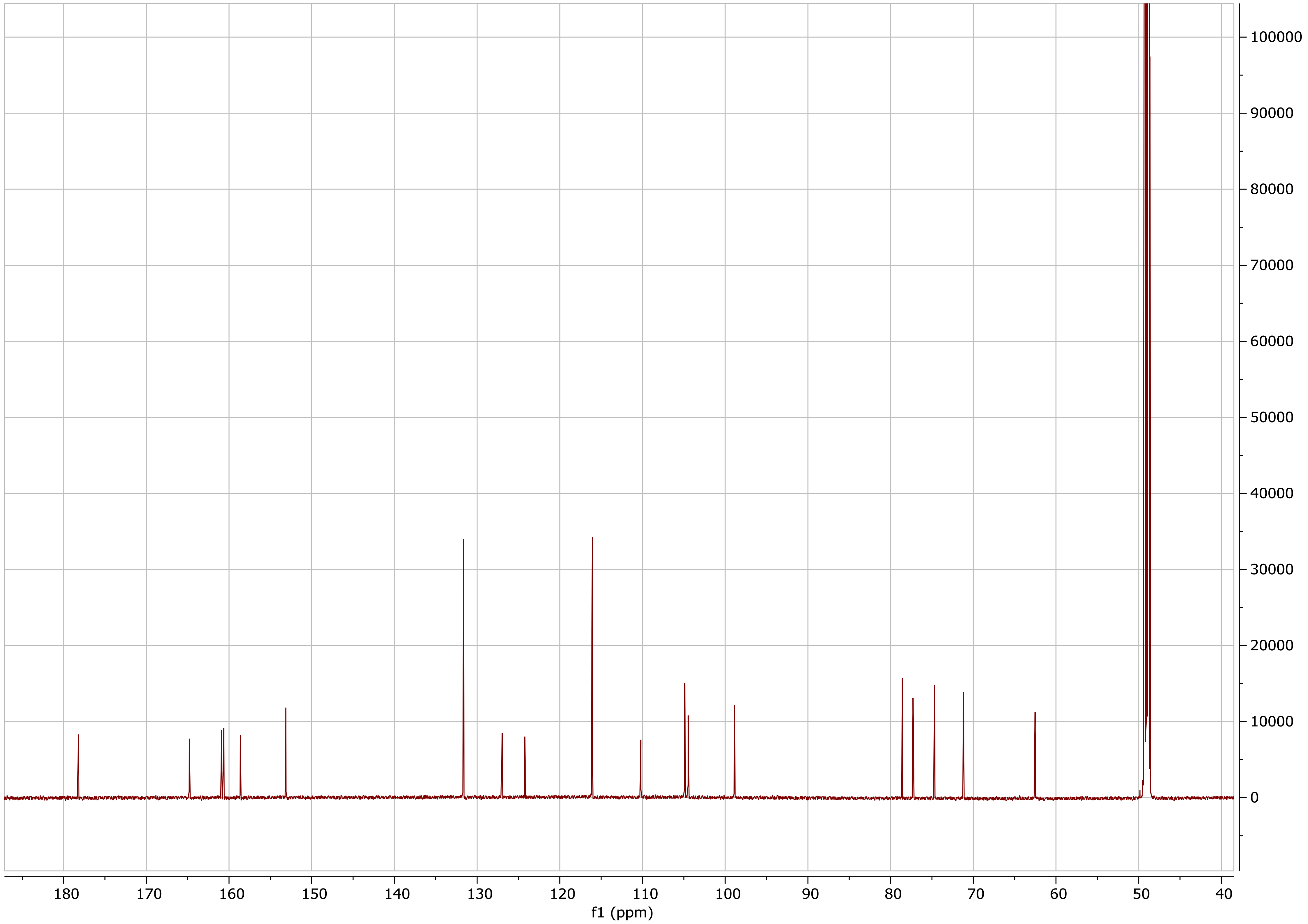

Figure S24. The ^{1}H NMR spectrum of **8** in methanol-d_4 + chloroform-d

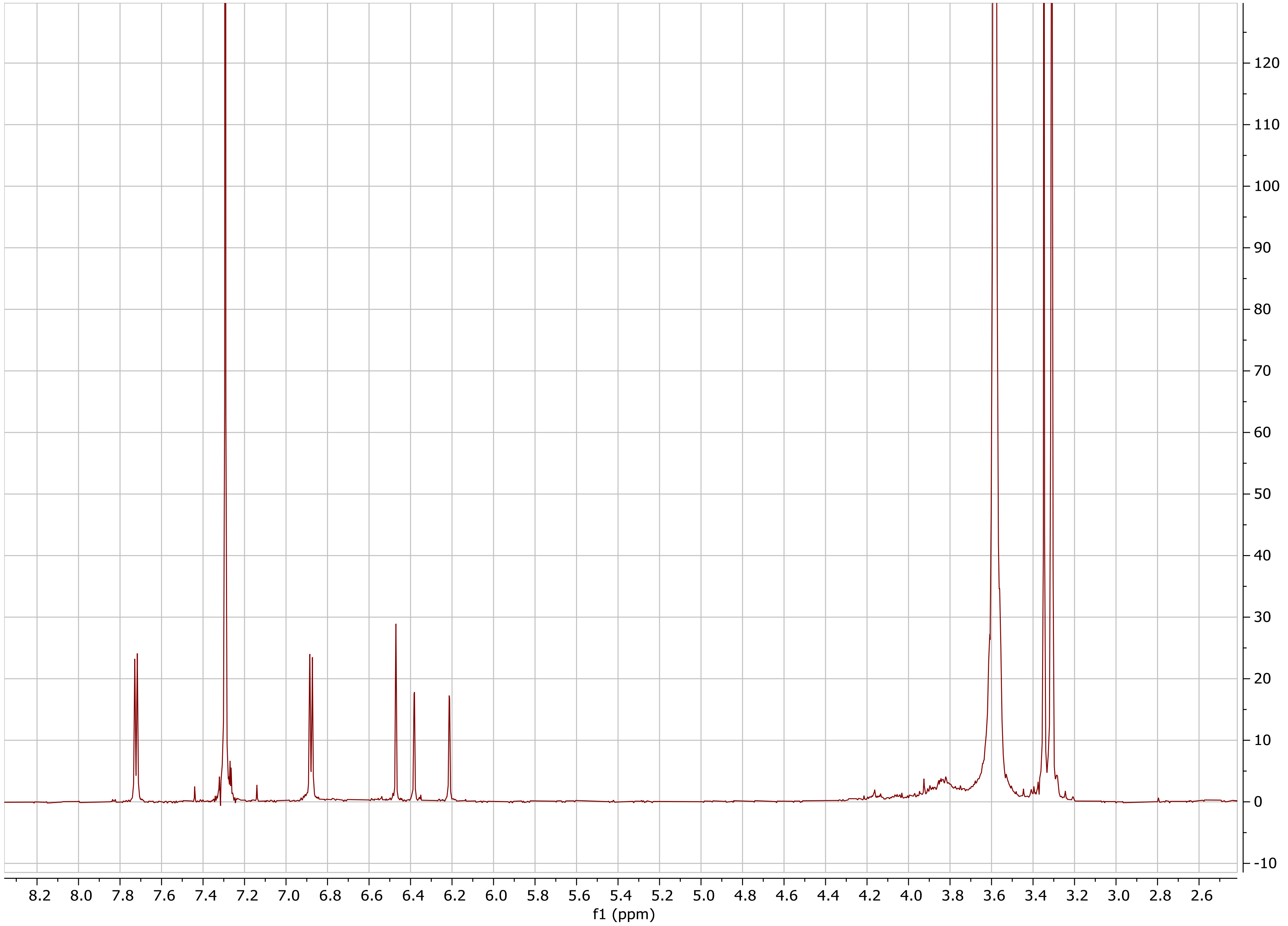

Figure S25. The ^{13}C NMR spectrum of **8** in methanol-d_4 + chloroform-d

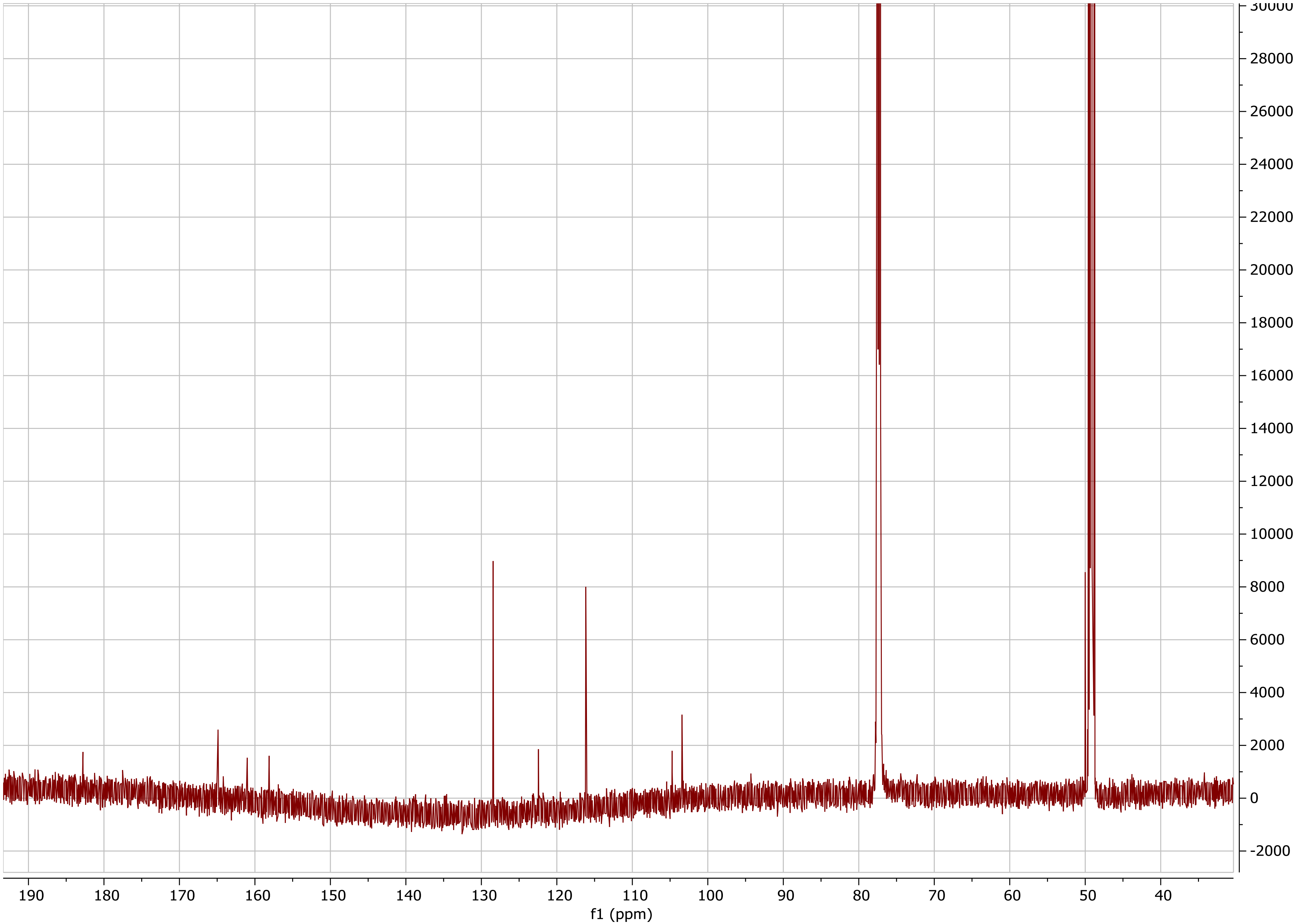

Figure S26. The ^{1}H NMR spectrum of **9** in methanol-d_4

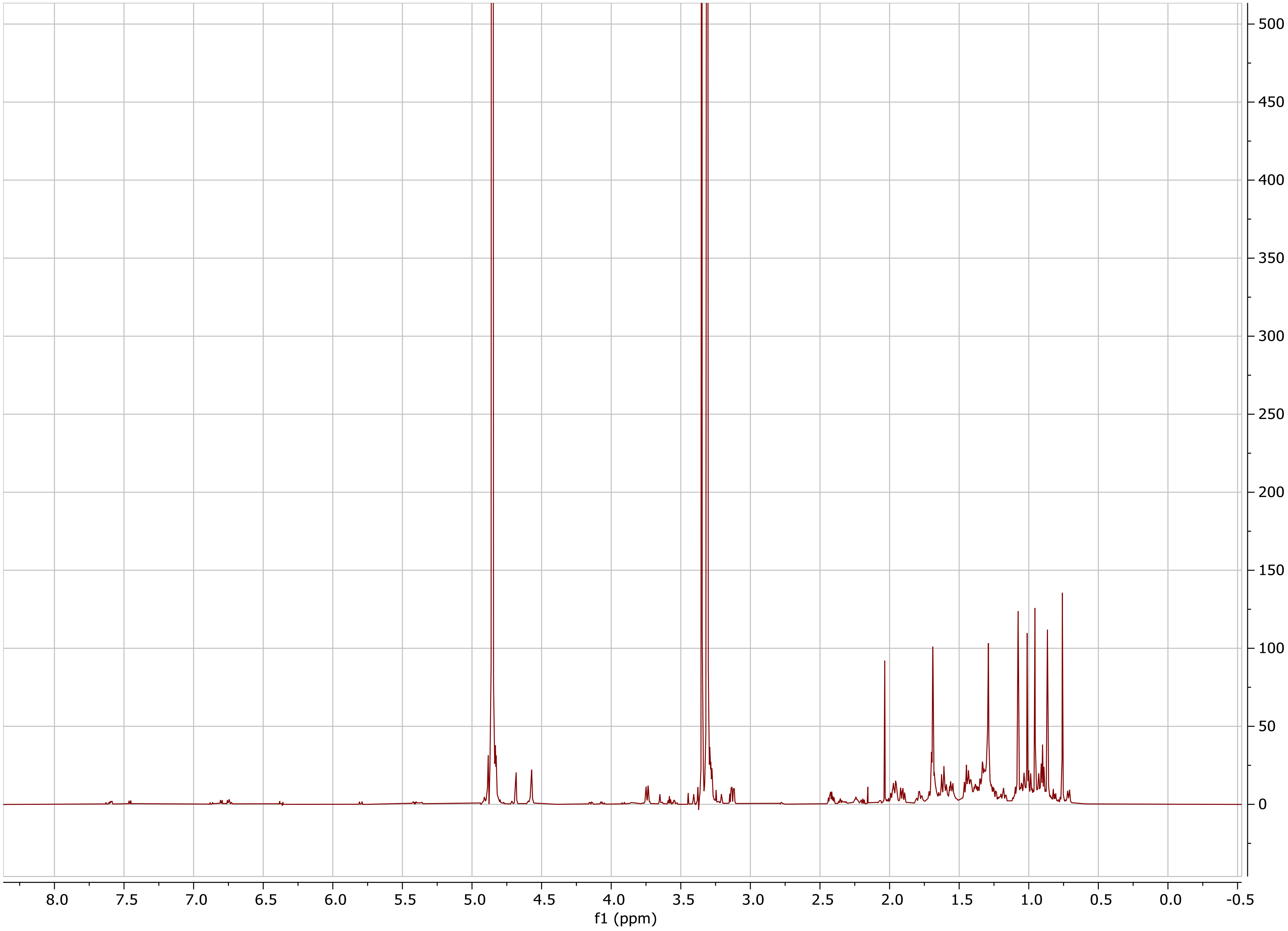

Figure S27. The ^{13}C NMR spectrum of **9** in methanol-d_4

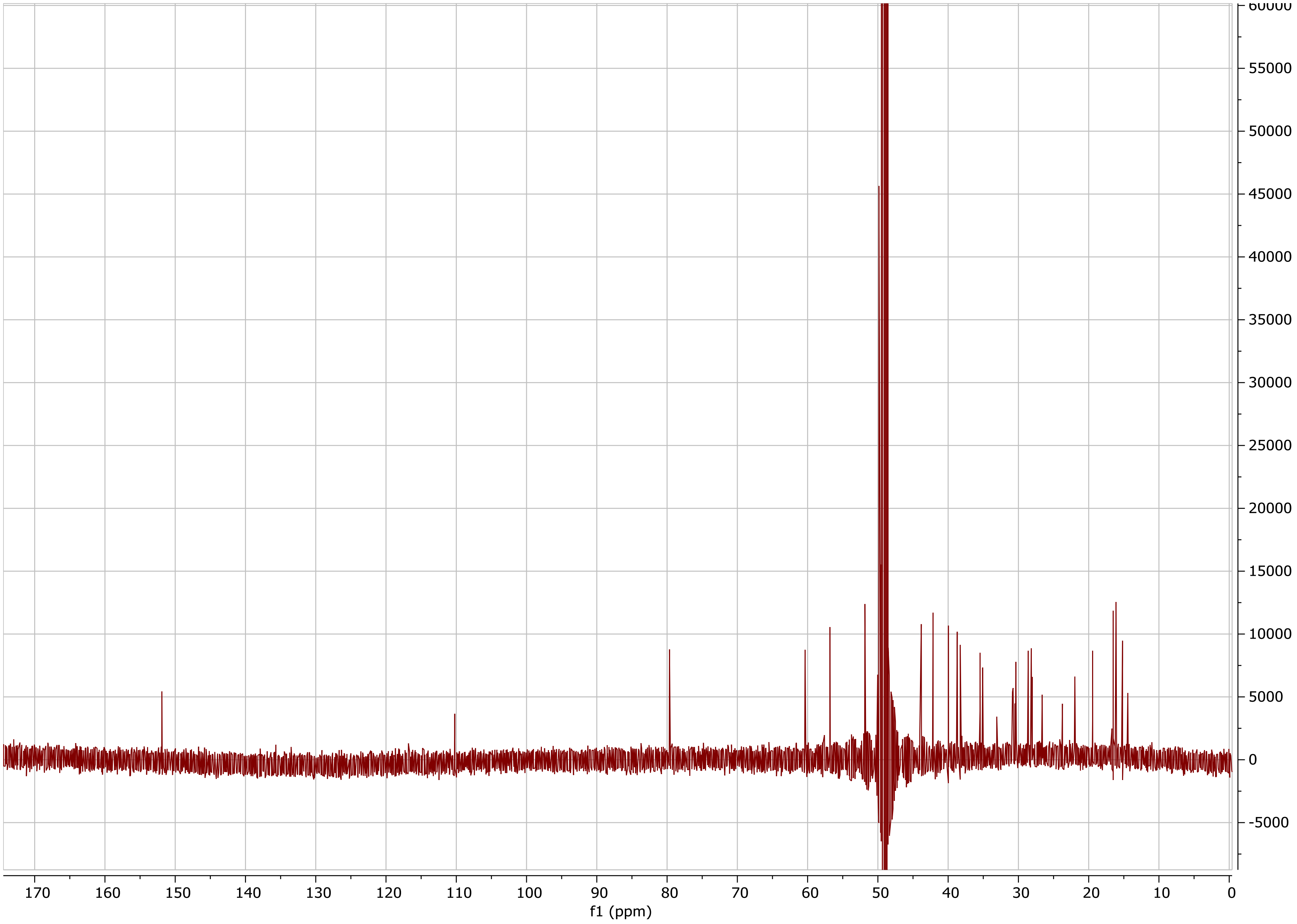

Figure S28. The ^{1}H NMR spectrum of **10** in methanol-d_4 + chloroform-d

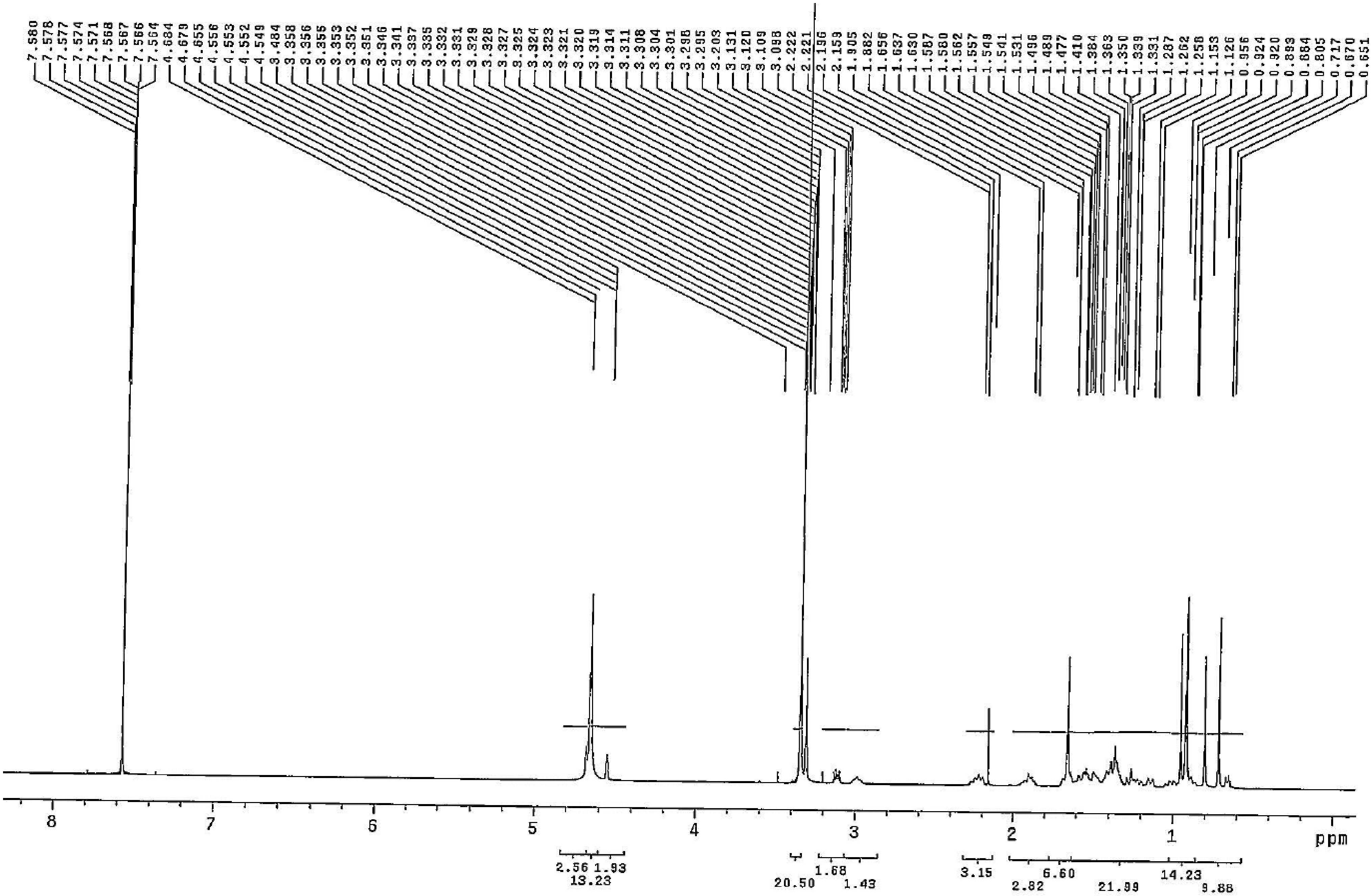

Figure S29. The ^{13}C NMR spectrum of **10** in methanol-d_4 + chloroform-d

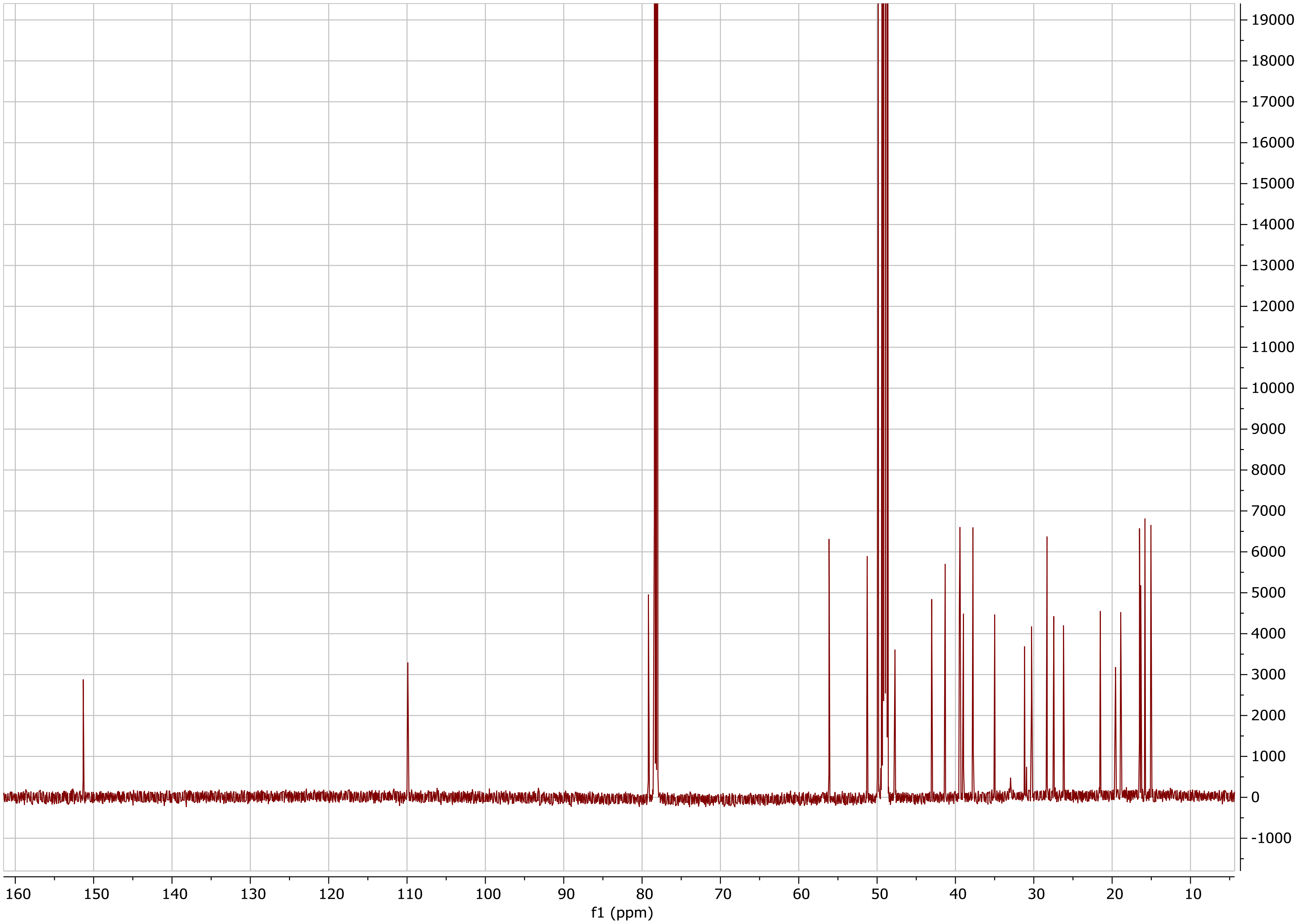

Figure S30. The ^{1}H NMR spectrum of **11** in chloroform-*d*

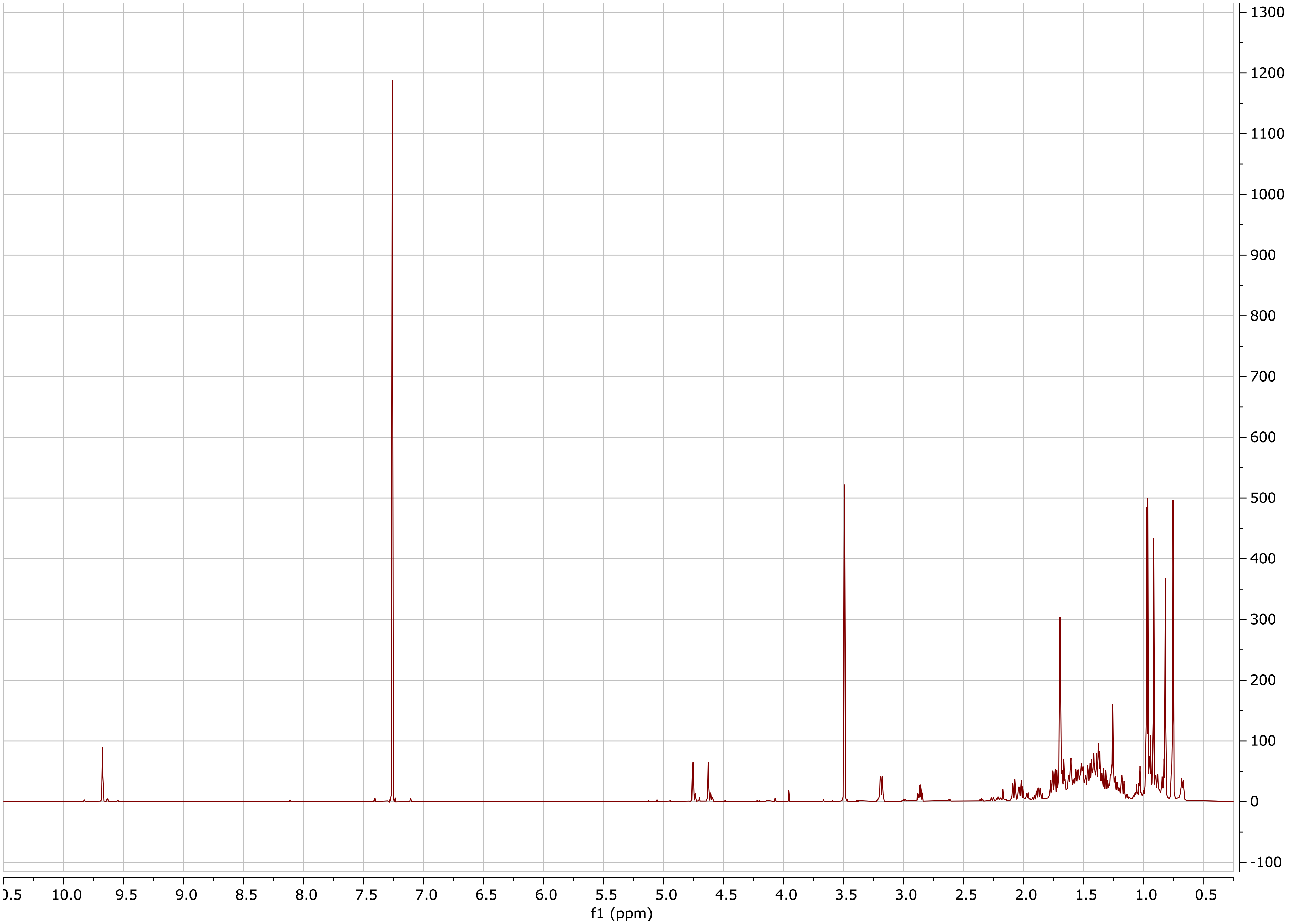

Figure S31. The ^{1}H NMR spectrum of **12** in pyridine-d_5

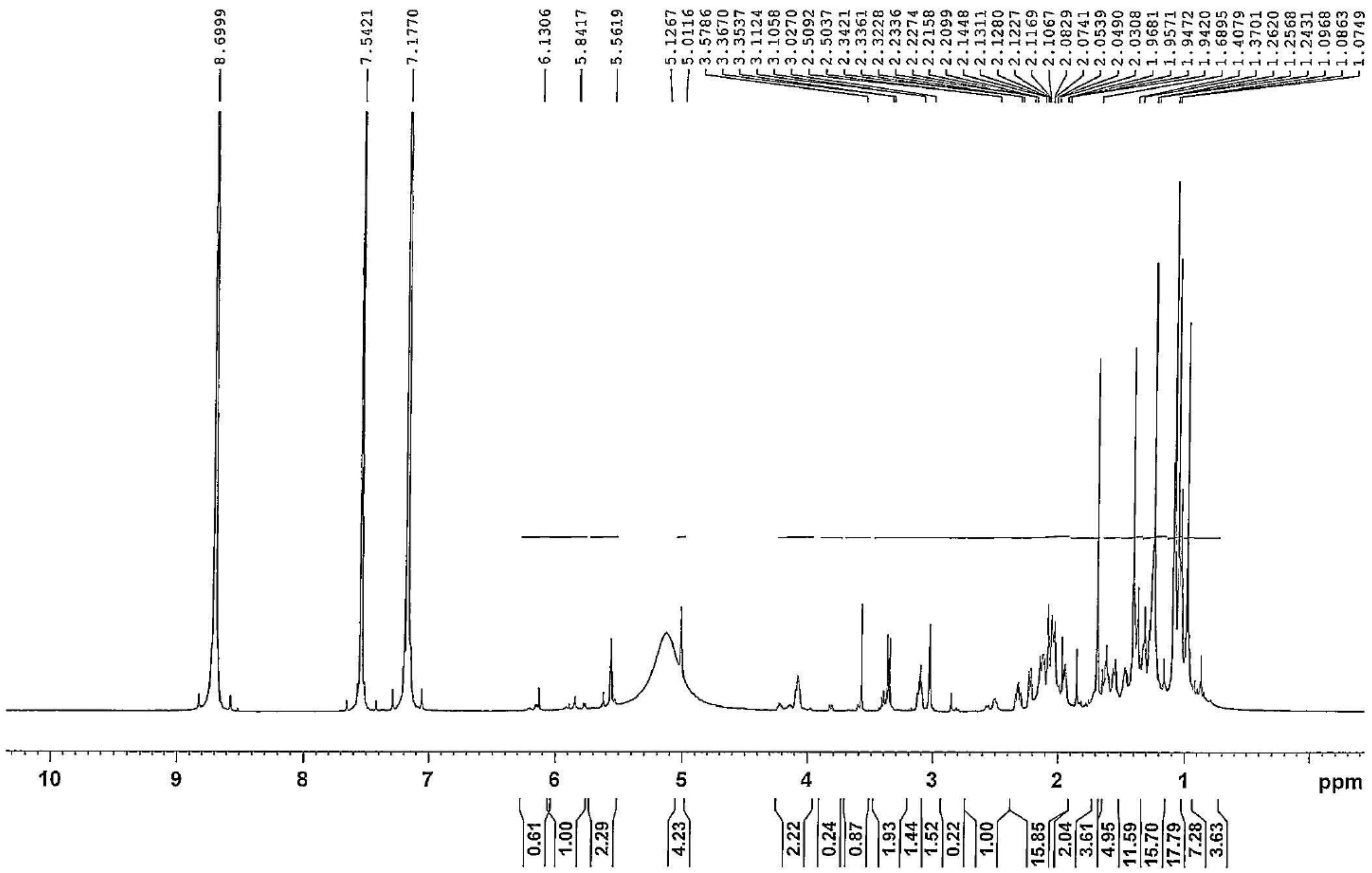

Figure S32. The ^{13}C NMR spectrum of **12** in pyridine-d_5

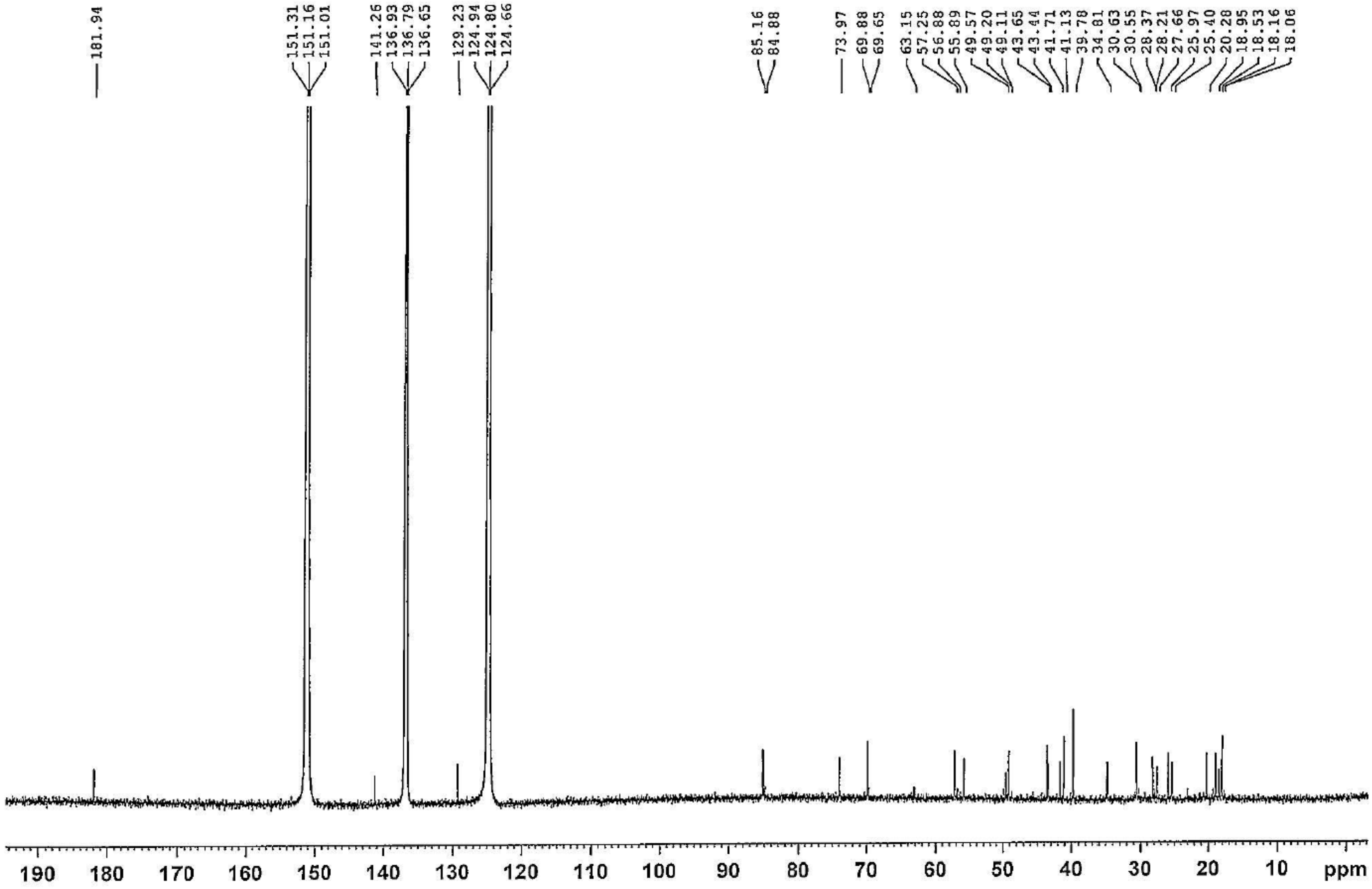

Supplement: File 1 — 1D and 2D NMR, HRMS, and ECD spectra of compound 1, 1H and 13C NMR spectra of 1a, and 1H NMR spectra of 1s and 1r. [file Beilstein_J_Org_Chem-16-3078-s001.pdf]
